# Supplementary material for: A multi-tiered workflow for examining organic acid profiles delineates tissue-specific changes in fatty acyl partitioning during aging
Source: Cell Rep Methods. 2026 Apr 21;6(5):101413. doi: 10.1016/j.crmeth.2026.101413 (PMC13198126; doi:10.1016/j.crmeth.2026.101413)
Supplement: Document S1. Figures S1 and S2 and Table S2 [file mmc1.pdf]

Cell Reports Methods, Volume *6*

Supplemental information

A multi-tiered workflow for examining organic acid profiles delineates tissue-specific changes in fatty acyl partitioning during aging

Zhiyang Zhou (周志阳), Chenyin Cao (曹晨音), Taochao Lu (陆陶超), Yuyuan Ruan (阮玉园), Bowen Li (李博文), Mingjun Cao (曹明君), Luyue Mo (莫璐月), Guanghou Shui (税光厚), and Sin Man Lam (林茜雯)

Supplemental Tables

Table S2. Percent recovery of representative organic acids from different subclasses in brain tissue matrix, related to Figure 3.

Mean % recoveries were calculated from averages of three independent experiments.

Class	*Representative species*	*% Recovery in brain tissue matrix*
SCFA	FA3:0	75.8
MCFA	FA14:0	85.1
LCFA	FA16:1(ω10)	89.1
OCFA	FA17:0	100.1
PUFA	FA20:4	116.4
	FA22:6	115.8
VLCFA	FA26:0	89.6
	FA28:0	93.4
	FA30:0	96.1
TCA	succinic acid	101.8
	fumaric acid	85.8
	malic acid	103.3
	alpha-ketoglutaric acid	111.8

SCFA: short-chain fatty acids; MCFA: medium-chain fatty acids; LCFA: long-chain fatty acids; OCFA: odd-chain fatty acids; PUFA: polyunsaturated fatty acids; VLCFA: very-long-chain fatty acids; TCA: tricarboxylic acids

Supplemental figures

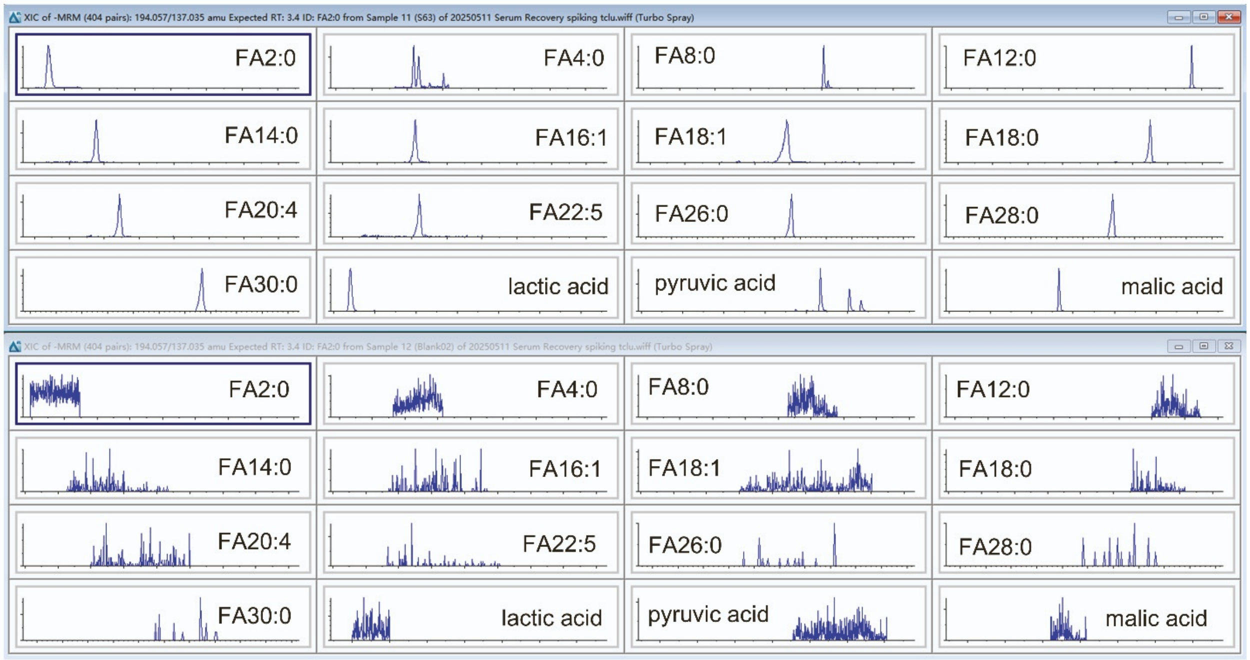

Figure S1. Carryover of individual organic acids under our liquid chromatographic gradient scheme, related to Figure 2. XICs of representative organic acids in reference standard mix (upper panel) and blank sample (lower panel) injected immediately after the reference standard mix showed negligible carryover under our liquid chromatographic gradient scheme**.**

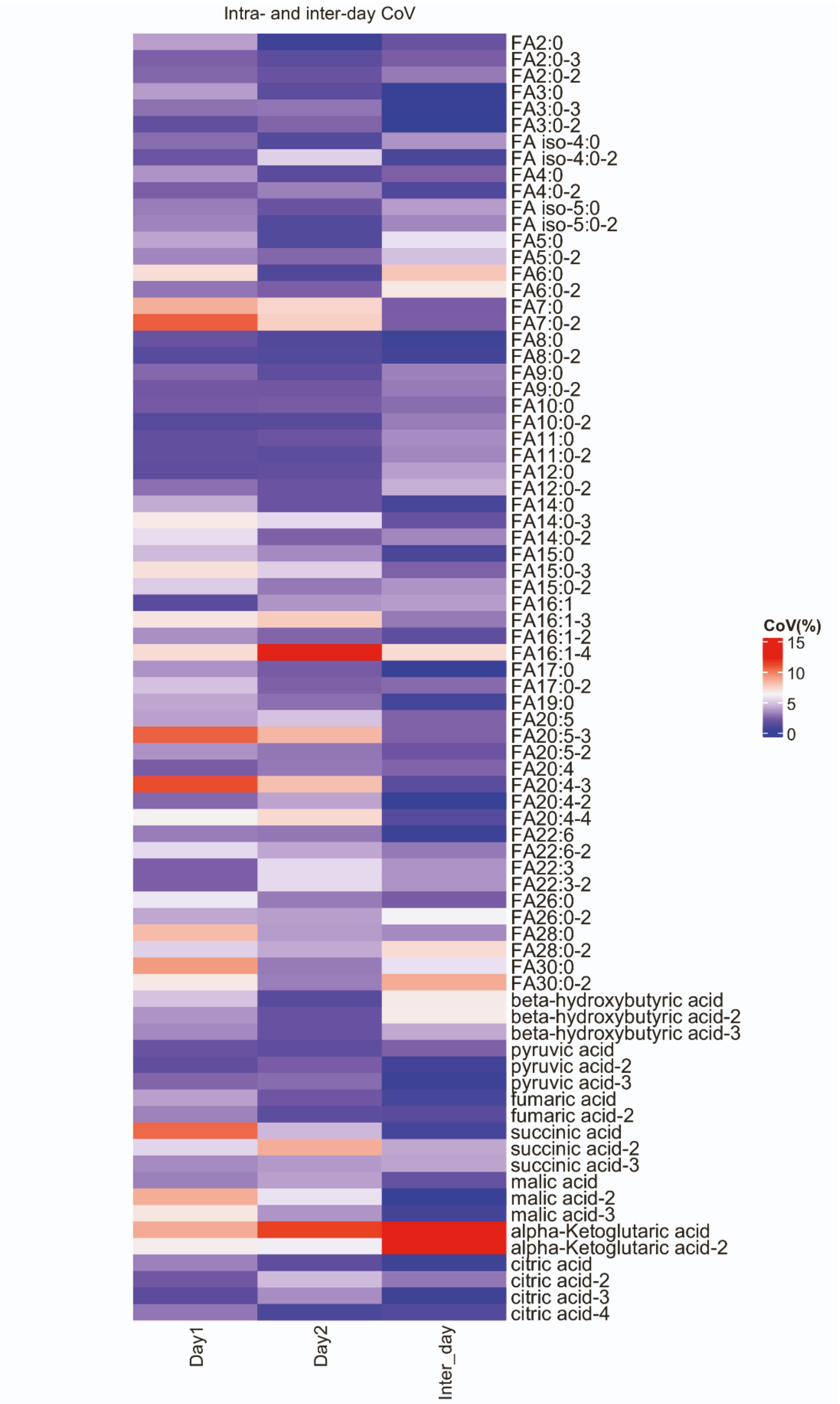

Figure S2. Intra-day and inter-day coefficient of variations, related to Figure 3. Heatmap summarizes the intra-day (three time-points on each day for two days, n=6 technical replicates) and inter-day (Day 1 vs Day 2, n=6 technical replicates) coefficient of variations (COVs) for individual organic acids quantified under our integrated methodology.
