# Supplementary material for: A multi-tiered workflow for examining organic acid profiles delineates tissue-specific changes in fatty acyl partitioning during aging
Source: Cell Rep Methods. 2026 Apr 21;6(5):101413. doi: 10.1016/j.crmeth.2026.101413 (PMC13198126; doi:10.1016/j.crmeth.2026.101413)
Supplement: Document S2. Article plus supplemental information [file mmc3.pdf]

Cell Reports Methods

Article

A multi-tiered workflow for examining organic acid profiles delineates tissue-specific changes in fatty acyl partitioning during aging

Graphical abstract

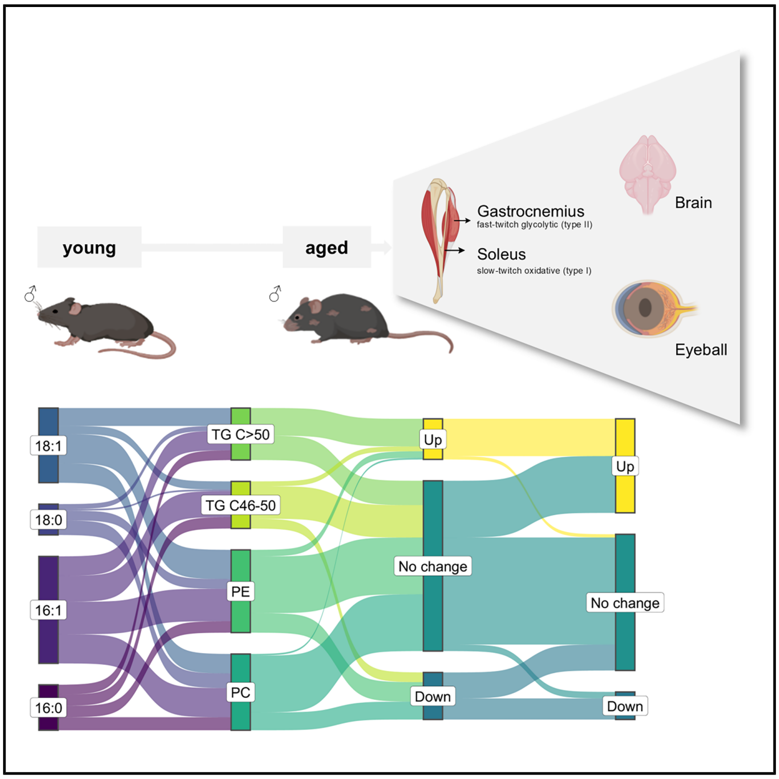

Highlights

- We develop a method to profile fatty acids from C2 to C34
- We provide the workflow to determine fatty acyl partitioning among lipid species
- We show that aged glycolytic muscles accrue odd-chain fatty acids in triacylglycerols
- Aging shifts oleic acid into diacylglycerols over phospholipids in skeletal muscle

Authors

Zhiyang Zhou (周志阳), Chenyin Cao (曹晨音), Taochao Lu (陆陶超), ..., Luyue Mo (莫璐月), Guanghou Shui (税光厚), Sin Man Lam (林茜雯)

Correspondence

shui_guanghou@gzlab.ac.cn (G.S.), smlam@lipidall.com (S.M.L.)

In brief

Zhou et al. develop a method to quantify the complete endogenous fatty acid spectrum and integrate the method into a multi-tiered workflow to determine fatty acyl partitioning across individual lipid species. Applied to aging tissues, it reveals how glycolytic and oxidative skeletal muscles rewire their fatty acyl partitioning across senescence.

Zhou et al., 2026, Cell Reports Methods 6, 101413
May 18, 2026 © 2026 The Author(s). Published by Elsevier Inc.
https://doi.org/10.1016/j.crmeth.2026.101413

CellPress

Cell Reports Methods

CellPress
OPEN ACCESS

Article

A multi-tiered workflow for examining organic acid profiles delineates tissue-specific changes in fatty acyl partitioning during aging

Zhiyang Zhou (周志阳),[1,6] Chenyin Cao (曹晨音),[2,3,4,6] Taochao Lu (陆陶超),[1,6] Yuyuan Ruan (阮玉园),[1] Bowen Li (李博文),[1] Mingjun Cao (曹明君),[2] Luyue Mo (莫璐月),[2,3] Guanghou Shui (税光厚),[2,3,4,*] and Sin Man Lam (林茜雯)[5,1,7,*]

[1]LipidALL Technologies Company Limited, Jiangsu Provincial Key Laboratory of Molecular Targets and Intervention for Metabolic Diseases, Changzhou 213022, China
[2]Institute of Genetics and Developmental Biology, Chinese Academy of Sciences, Beijing 100101, China
[3]University of the Chinese Academy of Sciences, Beijing 100049, China
[4]Guangzhou National Laboratory, Guangzhou, Guangdong 510005, China
[5]Guangzhou Institute of Cancer Research, the Affiliated Cancer Hospital, Guangzhou Medical University, Guangzhou 510095, China
[6]These authors contributed equally
[7]Lead contact
*Correspondence: shui_guanghou@gzlab.ac.cn (G.S.), smlam@lipidall.com (S.M.L.)
https://doi.org/10.1016/j.crmeth.2026.101413

MOTIVATION Comprehensive profiling of fatty acids across the full chain-length spectrum (C2–C34) and their esterification into different lipid species is hindered by the wide polarity disparity of these molecules. To address this, we developed an integrated, three-tiered workflow that quantifies the complete endogenous organic acid spectrum, enabling systems-level analysis of fatty acid metabolism and acyl partitioning in different biological contexts.

SUMMARY

Fatty acids (FAs), as the predominant organic acids, form a major component of the metabolome. We present a multi-tiered method that comprehensively captures FA diversity—including chain lengths (C2–C34), unsaturation, isomers, and endogenous forms—within a single biological specimen. This workflow quantifies the broadest range of free FAs reported to date. Integrated with two complementary tiers profiling the total FA pool from alkaline hydrolysis and esterified acyl compositions across lipid classes, our multi-tiered workflow enables the investigation of differential fatty acyl partitioning. Applying this platform to quantify >540 unique lipids (free and esterified forms) and polar carboxylic acids, we investigated FA remodeling in the brain, retina (eyeball), and skeletal muscles of young and aged mice. We found that aged glycolytic tissues preferentially partition odd-chain and diunsaturated FAs (with lower β-oxidizability) into triacylglycerols. Additionally, aging shifts the FA18:1 partitioning into diacylglycerols over anionic phospholipids, which may mitigate pro-aging lipid signatures in the skeletal muscle.

INTRODUCTION

Organic acids are, by definition, acidic compounds that contain at least one carboxylic acid functional group (–COOH) in their chemical structures and constitute a substantial portion of the human metabolome, with over 65% of the ~5,000 known endogenous human metabolites being broadly classified as organic acids.[1] Organic acids are predominantly represented by fatty acids (FAs), which make up a vast proportion of the endogenous organic acid pool, both in terms of numbers and abundances. FAs serve as important energy substrates to sustain cellular processes and are essential building blocks of glycerolipids, including neutral lipids and phospholipids.[2] FAs esterified into phospholipids modulate membrane property and fluidity, while the release of esterified polyunsaturated fatty acids (PUFAs) in response to external cues can produce signaling mediators that coordinate essential life processes.[3] As an example, we previously reported age-dependent temporal dynamics of esterified PUFAs in the neural membranes of Rhesus macaques.[4] The comprehensive analysis of free fatty acids (FFAs) is technically challenging owing to the large disparity in chemical polarity between short-chain fatty acids (SCFAs, C2–C6), medium-chain fatty acids (MCFAs, C8–C14), long-chain fatty acids (LCFAs, C16–C20), and very long-chain fatty acids (VLCFAs ≥ C22) that

© 2026 The Author(s). Published by Elsevier Inc.
This is an open access article under the CC BY license (http://creativecommons.org/licenses/by/4.0/).

makes satisfactory chromatographic separation difficult within one single injection. While preceding research has predominantly focused on even-numbered and long-chain FAs, emerging evidence highlights the unique biological roles of VLCFAs and odd-chain fatty acids (OCFAs). Accumulation of ceramides containing VLCFAs was shown to disrupt membrane integrity and trigger necroptosis,[5] while C28-C36 PUFAs fulfill important physiological roles in various mammalian tissues including the retina[6] and the brain.[7] Cohort studies also revealed inverse correlations between the levels of OCFAs esterified in plasma phospholipids and the risk of cardiovascular disease[8] and type 2 diabetes.[9] Apart from acyl chain lengths, the degree and position of unsaturation (i.e., C=C bonds) of the fatty acyl chains also substantially influence the physiochemical and functional properties of FAs, whether free or esterified. For instance, transgenic expression of *C. elegans fat-1*, which catalyzes the conversion of endogenous omega-6 PUFAs to omega-3 PUFAs, was found to ameliorate diet-induced metabolic dysfunction-associated steatotic liver disease (MASLD) in hamsters.[10] Finally, the endogenous forms of FAs, whether as FFAs or esterified into acyl constituents of distinct lipid classes, exert deterministic roles on the precise function of individual FAs in various physiological and pathological contexts, as extensively investigated by our group and others.[11–14]

Due to the chromatographic incompatibility of FAs across the wide polarity spectrum, preceding methods on FA analyses have mostly captured FAs of specific ranges with regard to acyl chain lengths, for example, C16–C23,[15] C14–C22,[16] as well as C10–C24.[17] Using twin derivatization strategy that leverages 5-dimethylamino-naphthalene-1 sulfonyl piperazine and diethylamino-naphthalene-1-sulfonyl piperazine, Jiang et al. quantified SCFAs, MCFAs, and LCFAs (C2–C24) within a single injection across a wide dynamic range, with a lower limit of quantification (LLOQ) ranging from 2 to 20 nM.[18] An integrated methodology to simultaneously quantify endogenous SCFAs, LCFAs, and VLCFAs (>C24) within a single injection, however, has been lacking thus far. As a result of the inherent volatility and poor retention of SCFAs under liquid chromatography (LC)-based separation, gas chromatography (GC) is conventionally used for the analyses of SCFAs.[19] Derivatization of SCFAs with 3-nitrophenylhydrazine (3-NPH) facilitates sensitive and reliable analyses of SCFAs,[20] which greatly enhances the ionization efficiency and improves LC-based chromatographic separation of carboxylates, while enhancing linearity and lowering the limits of detection (LODs) and quantification (LOQs).[21] Beyond SCFAs, the carboxyl groups of other carboxylic acids can be similarly derivatized with phenylhydrazines to their corresponding phenylhydrazones via nucleophilic addition, using water-soluble carbodiimides such as N-(3-dimethylaminopropyl)-N′-ethylcarbodiimide (EDC; Figure 1), among which 3-NPH exhibited superior reactivity and produced derivatized compounds of higher detection sensitivity.[21] Furthermore, fragmentation of the resultant phenylhydrazones under collision-induced dissociation (CID) involves losing the derivatized functional group incorporated, with the hydrocarbon (acyl) chains remaining intact. These chemical properties make 3-NPH derivatization ideal for tracing the metabolic flux of carboxylic acids at enhanced sensitivity. Indeed, we have broadly applied this technique to investigate metabolic flux of FA oxidation and subsequent channeling into the tricarboxylic acid (TCA) cycle.[22,23]

FA oxidation occurs primarily via β-oxidation in the mitochondrial matrix, which essentially breaks down acyl chains into smaller units (in the form of acetyl-CoAs) that enter the TCA cycle, generating energy to drive the electron transport chain for ATP production. Moreover, oxidation of OCFAs yields propionyl-CoAs, which can be converted into succinyl-CoAs that serve to replenish the TCA cycle substrates with anaplerotic intermediates, thereby improving mitochondrial energy metabolism.[24] In this work, we devised an integrated analytical platform that comprehensively measures the full FA spectrum in terms of acyl chain lengths (C2–C34), acyl chain unsaturation, positional isomers, and endogenous forms, as well as organic acids implicated in glycolysis and the TCA cycle within a single biological specimen. Our workflow accommodates the widest range of FA acyl chain lengths reported to date, and the derivation of esterified FA content enabled us to probe into the differential distributions of individual FAs among various lipid classes across different tissues. Mitochondrial energy metabolism is known to decline with enhanced metabolic stress across aging. Thus, we applied our multi-tiered workflow to investigate age-associated perturbations in cellular energy metabolism, reflected by alterations in endogenous organic acid profiles of the skeletal muscles, brain, and retina of young (6-month-old) and old (23-month-old) mice (human equivalent ages of ≈20–30 years and ≈56–69 years, respectively).[25]

RESULTS

A multi-tiered workflow for integrated analysis of endogenous organic acid profiles

We devised an integrated workflow to comprehensively quantify endogenous organic acids, including C2–C34 FAs and polar carboxylic acids implicated in glycolysis and the TCA cycle, within a single injection (Figure 1, branch C). When required, the methodology can be combined with two additional tiers of FA profile analysis within one single biological sample. These involve quantitative lipidomics that extensively characterizes the profiles of esterified FAs in different glycerolipid classes and species (Figure 1, branch A), and the use of alkaline hydrolysis to quantitatively profile the cumulated sums (free + esterified forms) of individual FAs (i.e., total FAs) present within individual samples (Figure 1, branch B). The content of esterified forms of individual FAs can be determined by subtracting free FA content from the total FA content (i.e., esterified FA = total FA − free FA), using quantitative results from branches C and B, respectively. In this manner, our multi-tiered workflow allows us to calculate the fractional distributions of individual esterified FAs among different glycerolipid classes in various tissues across aging, which essentially reflects changing FA remodeling of tissue senescence. We applied the multi-tiered workflow developed to analyze the comprehensive organic acid profiles from the brain, eyeball, and skeletal muscles (gastrocnemius [Gas] and soleus) from male mice aged 6 and 23 months. In all, the single-tiered arm (branch C) detected and quantified 116 unique organic acids from the various murine tissues, which consisted of 7 SCFAs, 6 MCFAs, 38 LCFAs, 50 VLCFAs, and 15 polar carboxylic acids relevant to glycolysis

CellPress
OPEN ACCESS

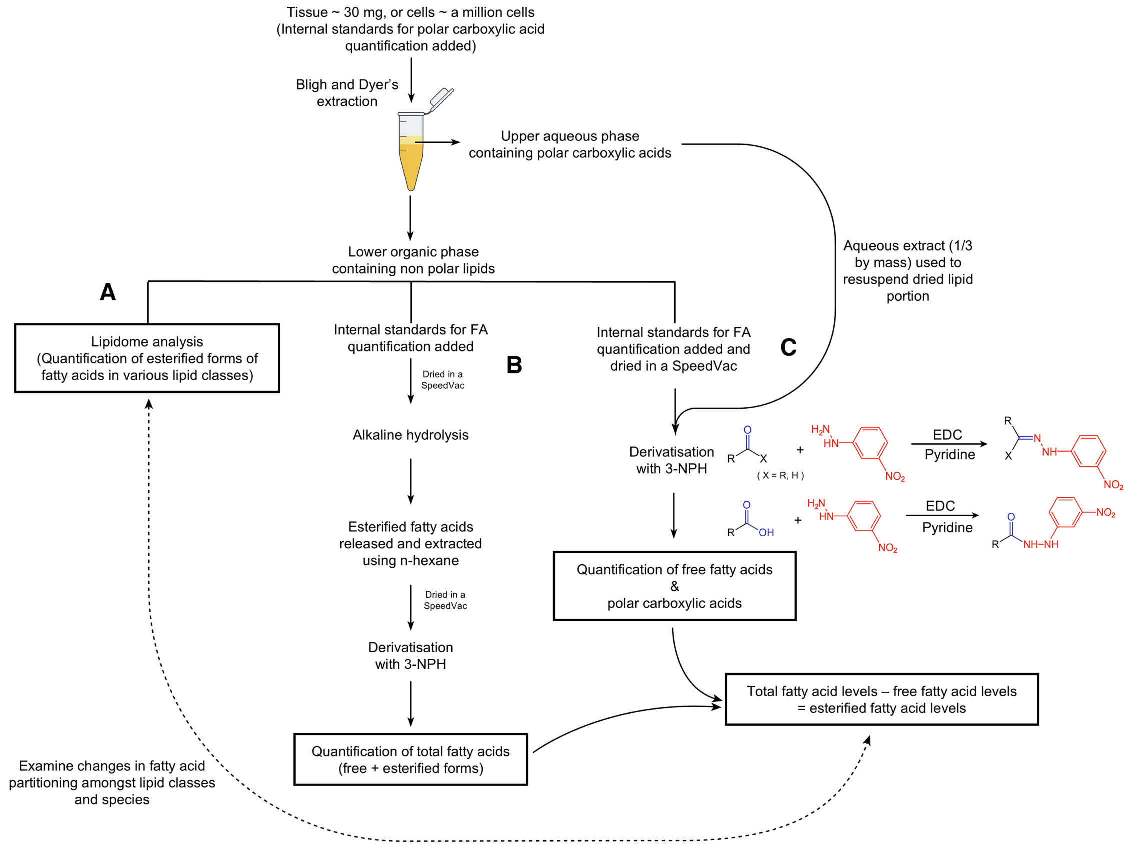

Figure 1. Schema of three-tiered analytical workflow

Tissues (≈30 mg) were subjected to modified Bligh and Dyer's extraction, and deuterated internal standards for polar carboxylic acid quantification were added at lipid extraction. The lower organic extract containing nonpolar lipids was split into three portions for separate analyses under each of the three tiers. Under branch C, a portion of the organic extract was combined with the upper aqueous phase from Bligh and Dyer's extraction containing polar carboxylic acids, which constitutes an integrated workflow to comprehensively capture endogenous organic acids including C2–C34 FAs and polar carboxylic acids participating in glycolysis and TCA cycle, within a single injection. Deuterated standards for FA quantification were added prior to derivatization with 3-NPH, and organic acids were detected as their nitrophenylhydrazones, using targeted MRM approaches. Branch B utilizes alkaline hydrolysis to quantify the cumulated sums (free + esterified forms) of individual FAs (i.e., total FAs) present within the biological tissues. Esterified FA levels can be obtained by subtracting free FA levels (branch C) from total FA levels (branch B). Branch A refers to quantitative lipidomics that extensively characterizes the profiles of esterified FAs in different glycerolipid classes and species. Finally, by taking the fractions of individual glycerolipids containing a designated FA to the total esterified levels of that same FA, the partitioning of FAs into different glycerolipid classes and species across different biological conditions can be compared.
FA, fatty acid; TCA, tricarboxylic acid; MRM, multiple reaction monitoring; 3-NPH, 3-nitrophenylhydrazine.

and the TCA cycle. Leveraging the three-tiered approach on a single biological specimen, we quantified a repertoire of >540 unique lipids and polar carboxylic acids in murine tissues, inclusive of the various endogenous forms (esterified or free) of individual FAs.

Satisfactory chromatographic separation of FA isomers across a wide polarity spectrum

In order to accommodate the elution of MCFAs and VLCFAs within a single injection, we improved upon our previous chromatographic gradient that accommodates only SCFAs and MCFAs.[22] Under the new chromatographic conditions, polar carboxylic acids and FAs ranging from C2 to C34 were well separated with satisfactory peak shapes, with more hydrophobic species eluting at later retention times (Figure 2A). Individual members of each FA subclass displayed progressive elution, while the ω-3 and ω-6 isomers of PUFAs, including FA 18:3, FA 20:4, and FA 22:5, achieved baseline separation under the current gradient (Figure 2B). In addition, our LC scheme exhibited no appreciable carryover across successive injections (Figure S1), which ensures accurate quantification of the various organic acid species and isomers in high-throughput analyses.

LOD, LOQ, linearity, recovery, and precision of measurements

We examined the LOD, LOQ, and linearity of our methodology, leveraging serially diluted solutions of standard compounds with concentrations ranging from 0.0005 to 0.25 mg/L. The standard error of response (σ) and regression coefficient (S) of least-square linear regressions based on peak areas at individual concentrations, expressed in mmol/L, were used for LOD and LOQ determination, where LOD = 3.3σ/S and LOQ = 10σ/S. Final

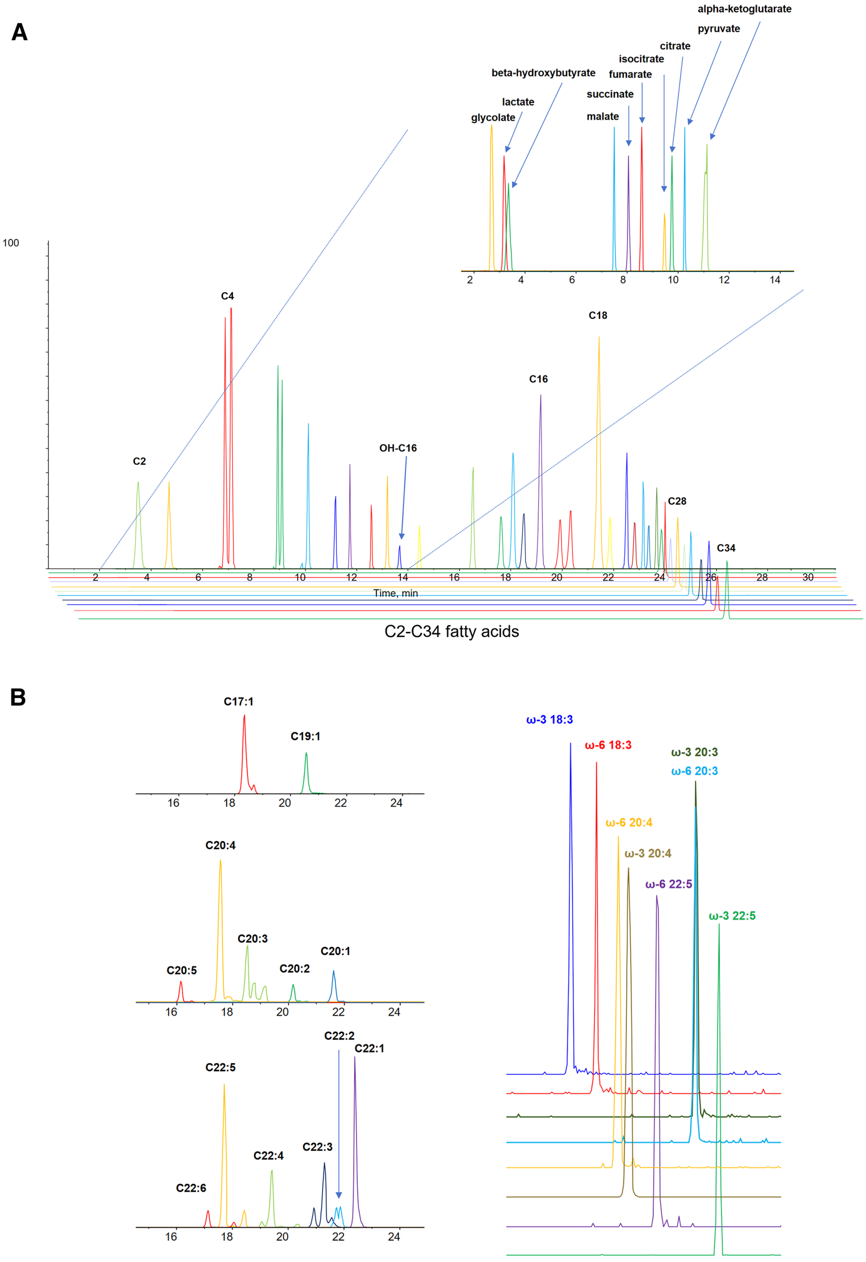

Figure 2. Extracted ion chromatograms of individual organic acids

(A) Extracted ion chromatograms (XICs) of individual organic acids, comprising C2–C34 FAs and polar carboxylic acids involved in glycolysis and the TCA cycle.

(B) Left: individual FAs of the same series (e.g., 20:5, 20:4, 20:3, 20:2, and 20:1; and C22:6, C22:5, C22:4, C22:3, C22:2, and C22:1) displayed progressive elution according to decreasing polarity as the degree of unsaturation decreased or the length of hydrocarbon chain increased (e.g., C17:1 and C19:1). Right: ω-3 and ω-6 isomers of FA 18:3 and FA 22:5 exhibited satisfactory separation under our gradient program.

LODs and LOQs of individual organic acids are expressed in pmol (Table S1 and Figure 3). Our methodology displayed satisfactory sensitivity for the full range of organic acids investigated, with all analytes attaining LODs < 11 pmol (Figure 3 and Table S1). In particular, our method displayed superior sensitivity for VLCFAs, LCFAs, and MCFAs, for which the individual LODs attained fmol ranges (e.g., LODs for FA 30:0, FA 28:0, and FA 26:0 at 24.8, 53.1, and 18.5 fmol, respectively). The sensitivity for SCFAs was inferior compared to longer-chain FAs due to our choice of resuspension solvent (i.e., methanol) that best accommodates the full ranges of FAs under investigation, which may be less than ideal for SCFAs. Linearity ranges were assessed using $R^2 \geq 0.98$ as a cutoff based on least-squared linear regressions of individual analytes (Table S1). For polar carboxylic acids that displayed wider ranges in terms of endogenous abundances, such as lactic acid and pyruvic acid, the availability of multiple reaction monitoring (MRM) pairs with varying linear ranges allowed us to select the specific MRM pairs for quantification in distinct biological matrices, which ensured that our methodology effectively accommodates wide endogenous concentration ranges of various organic acids. Recovery was calculated in the brain tissue matrix as quantitated fold increase based on a 3-fold spiking relative to a 1-fold spiking after subtracting the endogenous backgrounds, i.e., % recovery as ([(S3-S0)/(S1−S0)]/3×100%), where S0, S1, and S3 denote quantified levels in unspiked brain tissue homogenate, brain tissue homogenate with one portion of reference compound mix added, and brain tissue homogenate with three portions of reference compound mix added, respectively. The reference compound mix comprises representative species from the subclasses SCFA (C3:0), MCFA (C14:0), LCFA (C16:1), OCFA (C17:0), PUFA (C20:4 and C22:6), VLCFA (C26:0, C28:0, and C30:0), and TCA (succinic acid, fumaric acid, malic acid, and alpha-ketoglutaric acid) (Table S2). Our integrated methodology achieved satisfactory recoveries (75.8%–116.4%) for all subclasses of organic acids examined (Table S2). The precision of quantification was calculated as the intra-day and inter-day coefficients of variations (COVs) by preparing and analyzing aliquots of a mixture of reference standards every 4 h within one day (six technical replicates) and across two consecutive days (six technical replicates), respectively (Figure S2). The high precisions of analytes reflect good chemical stability of the organic acid derivatives, which qualifies our methodology for large-scale, high-throughput analyses.

CellPress
OPEN ACCESS

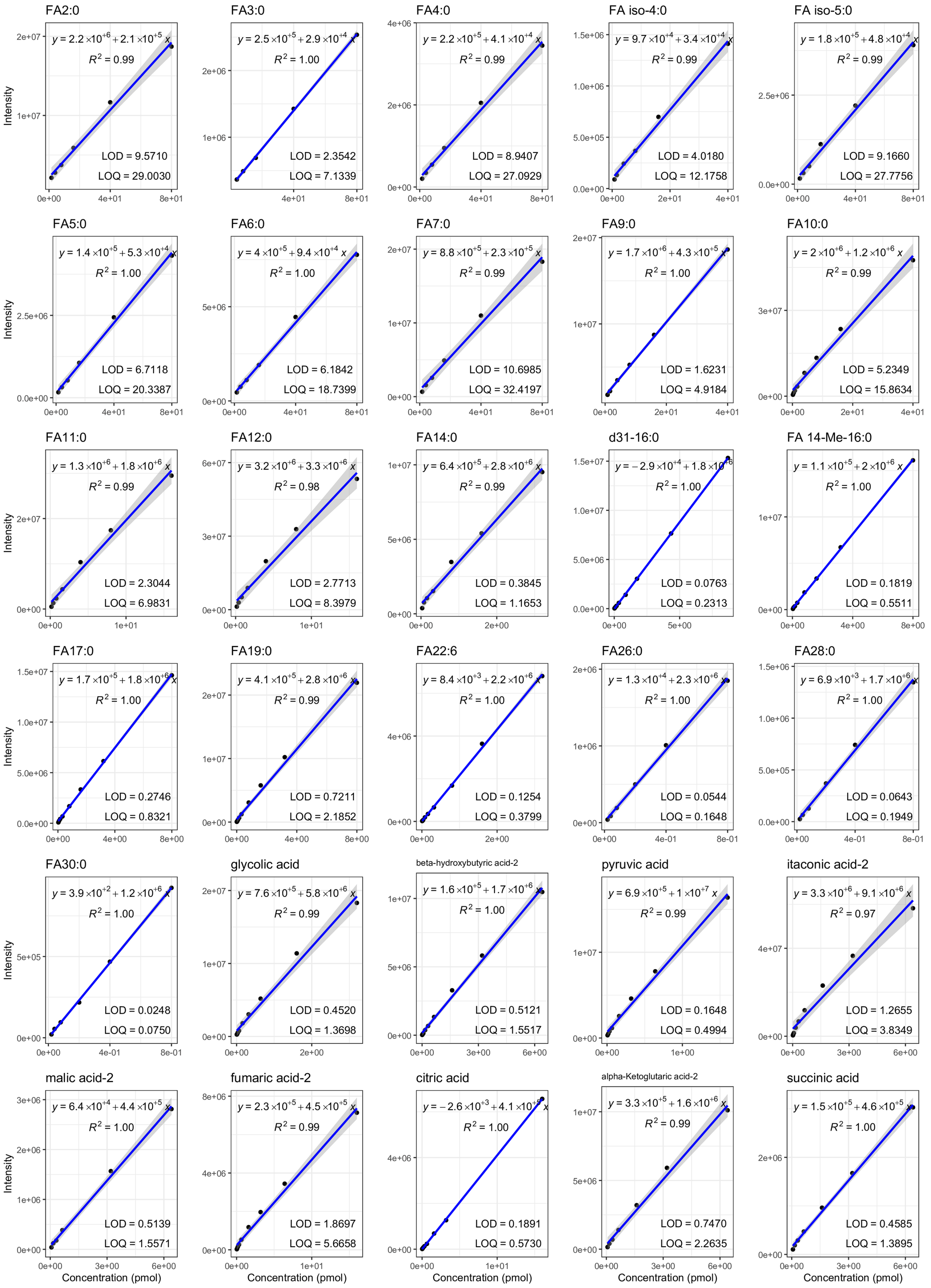

(legend on next page)

In vitro validation using etomoxir-treated C2C12 myotubes

To validate the biological utility of our multi-tiered methodology for examining differential fatty acyl partitioning among various glycerolipid classes, we treated C2C12 myocytes with etomoxir, an oxirane carboxylate that irreversibly inhibits mitochondrial carnitine palmitoyltransferase I (CPT-1), which preferentially transports long-chain acyl-CoAs (C16–C18) into the mitochondria for β-oxidation.[2] Etomoxir-mediated inhibition of CPT-1 was reported to trigger accumulation of intra-cellular lipids.[26] The perturbed FA partitioning of esterified long-chain acyls (C16:0, C16:1, C18:0, and C18:1) upon treatment of myotubes with etomoxir is summarized as a Sankey plot in Figure 4A. Aligned with previous reports, we observed increased partitioning of long-chain fatty acyls into triacylglycerols (TGs), which was enhanced with increasing concentrations of etomoxir from 40 μM to 80 μM (Figure 4). We noted, however, a small but significant decrease in the partitioning of esterified C16:0 into short-to-medium-chain TGs (C46–C50) at 40 μM etomoxir, indicating that the etomoxir-mediated inhibition of CPT-1 impedes β-oxidation of long-chain acyl-CoAs that are preferentially channeled into long-chain (C > 50) TGs in myotubes. Interestingly, we also observed reduced partitioning of esterified C16:0 and C18:0 into polyunsaturated phosphatidylcholines (PCs) and phosphatidylethanolamines (PEs) (PUFA-PCs and PUFA-PEs, respectively) in etomoxir-treated myotubes (Figure 4B).

Age-associated changes in endogenous lipid and organic acid profiles across tissues

We then applied our multi-tiered approach to examine senescence-associated changes in the lipid and organic acid profiles of the brain, eyeball, and skeletal muscles of young (6-month-old) and old (23-month-old) C57BL/6N male mice. The Gas muscle predominantly comprises fast-twitch glycolytic (type II) muscle fibers, while the soleus is principally composed of slow-twitch oxidative (type I) muscle fibers (Figure 5A). An UpSet plot was constructed to illustrate the number of age-associated metabolite changes conserved across the tissues examined (Figure 5B). The eyeball displayed the largest number of overlapping senescence-associated metabolites with other tissues (e.g., the Gas and the brain) (Figure 5B). Among skeletal muscles, the number of metabolites significantly altered with age was 3-fold higher in Gas compared to the soleus, which might be explained by prior observation that the Gas muscle appears to age and deteriorate at a quicker pace than the soleus muscle, with earlier onset of muscle atrophy and functional decline.[27] Across all four tissues examined, bis(monoacylglycero)phosphates (BMPs) were found to be consistently and significantly increased with age (Figure 5C), which corroborated preceding work reporting tissue-wide accumulation of BMPs during mouse aging. BMPs were previously shown to accumulate greatly in the skeletal muscles, brain, heart, kidney, liver, spleen, testes, and adipose of 24-month-old male C57BL/6J mice relative to 3-month-old mice, which the authors attributed to the enhanced lysosomal activity.[28]

Accumulation of esterified OCFAs and diunsaturated FAs in the eyeball and Gas muscle with age

The accumulation of esterified OCFAs, including FA 17:2, FA 17:1, FA 19:2, and FA 19:1, in the eyeball and Gas muscle with age was particularly evident (Figure 6A). Fractional distributions of esterified OCFAs among TGs were elevated in the eyeball and Gas muscle of old mice, indicating that esterified OCFAs were increasingly directed to odd-chain TGs during aging. Indeed, TGs displayed stark accumulations in both the eyeball and Gas muscle of old mice (Figures 6A and 6B). While sphingomyelins (SMs) comprising OCFAs, such as SM 33:1;O2, were also elevated in old mice, this did not contribute significantly toward the content of esterified OCFAs measured in our assays, because the N-acyl bond of sphingolipids is generally resistant to alkaline hydrolysis.[29] Besides OCFAs, the esterified levels of numerous diunsaturated FAs, including FA 18:2, FA 16:2, and FA 14:2, were also significantly increased in the eyeball and Gas muscle of aged mice (Figures 6A and 5C).

Age-associated reductions in very long-chain PUFAs in the eyeball

The eyeball of aged mice also exhibited distinct reductions in both free and esterified levels of very long-chain PUFAs (VLC-PUFAs), such as FA 24:5, FA 26:5, FA 26:6, FA 24:6, and FA 32:6 (Figure 6C). These observations are in agreement with a previous report on substantial diminutions in VLC-PUFAs (C32–C34) in the whole retina and retinal pigment epithelium of human donors with age.[30] VLC-PUFAs within the retina likely contribute positively toward retinal function, as the dietary supplementation of FA 22:6 was shown to protect the retina of aged mice from age-associated functional degeneration.[31,32] Decreased levels of both free and esterified VLC-PUFAs with age indicate that VLC-PUFA esterification into lipid membranes is likely unaltered with age and that these reductions might instead be ascribed to dietary changes, attenuated biosynthesis, or enhanced breakdown from β-oxidation or oxidative stress. Indeed, preceding work showed that labeled FA 22:6 was effectively incorporated into lipids of the aged retina.[33] Thus, reductions in esterified VLC-PUFAs with age likely stem from abated bioavailability of VLC-PUFAs instead.

Senescence-associated changes in organic acid profiles of oxidative (soleus) versus glycolytic (Gas) skeletal muscles

Both the Gas and soleus muscles displayed stark accumulation of esterified FA 18:1 in aged mice, with concomitant accumulation of phosphatidylinositols (PIs), PCs, and diacylglycerols

Figure 3. Least-squared linear regression models of individual organic acids for LOD and LOQ determination

Least-squared linear regression models were used to calculate LODs and LOQs. The standard error of response (σ) and regression coefficient (*S*) of least-square linear regressions based on peak areas at individual concentrations, expressed in mmol/L, were used for LOD and LOQ determination, respectively, where LOD = $3.3\sigma/S$ and LOQ = $10\sigma/S$. Final LODs and LOQs of individual organic acids are expressed in pmol.

LOD, limit of detection; LOQ, limit of quantification.

See also Table S1.

CellPress
OPEN ACCESS

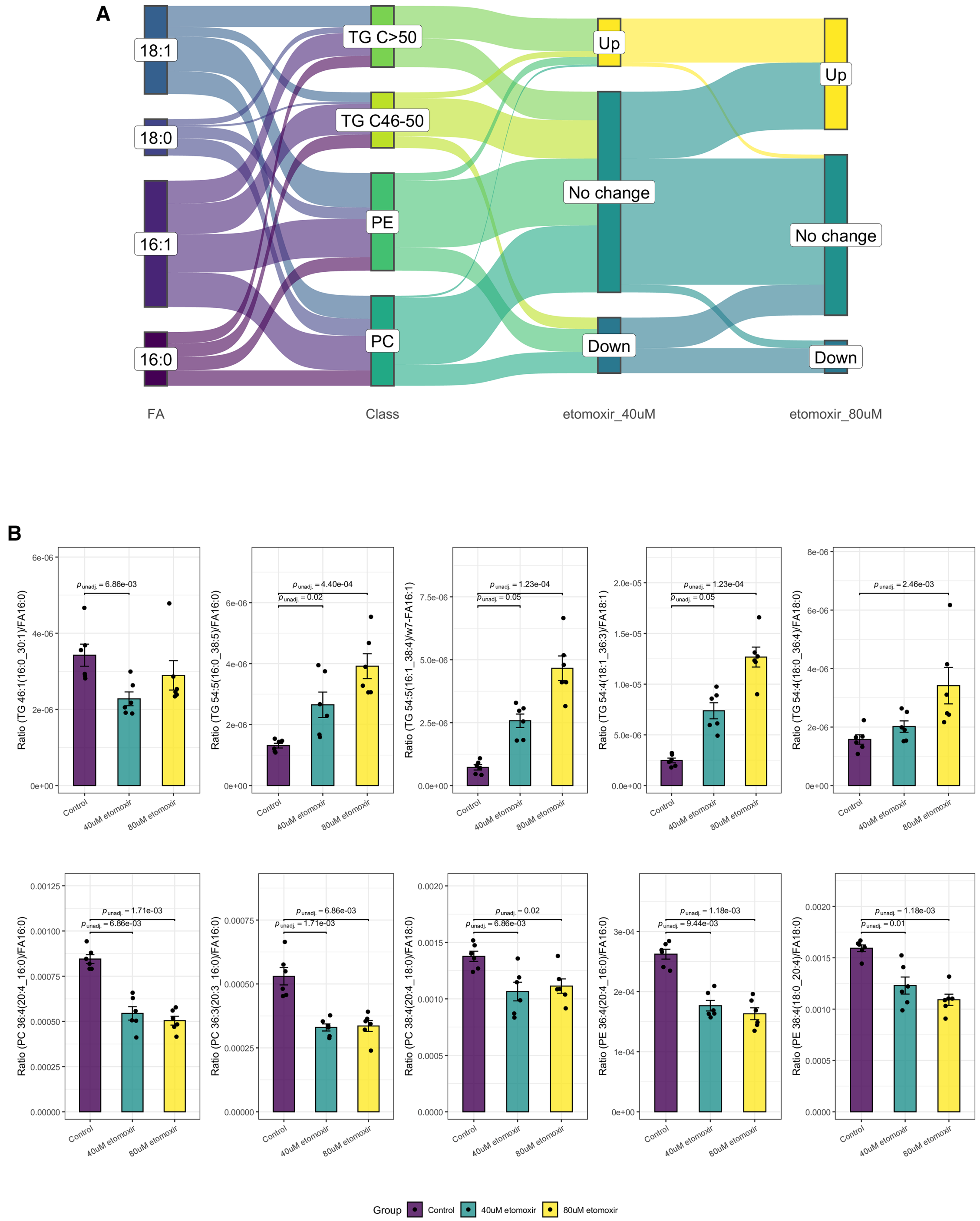

Figure 4. *In vitro* validation of multi-tiered methodology using etomoxir-treated myotubes

(A) Sankey plot illustrating changes in the partitioning of esterified long-chain acyl-CoAs including C16:0, C16:1, C18:0, and C18:1 into long-chain TGs (C > 50), short-to-medium-chain TGs (C45–C50), PCs, and PEs in C2C12 myotubes in the control/untreated group (n = 6), the group treated with 40 μM etomoxir (n = 6), and the group treated with 80 μM etomoxir (n = 6).

(B) Bar plots displaying changes in fatty acyl partitioning into TGs, PCs, and PEs with etomoxir treatment. p values from Student's t test are displayed. Error bars represent standard errors of the mean (SEM).

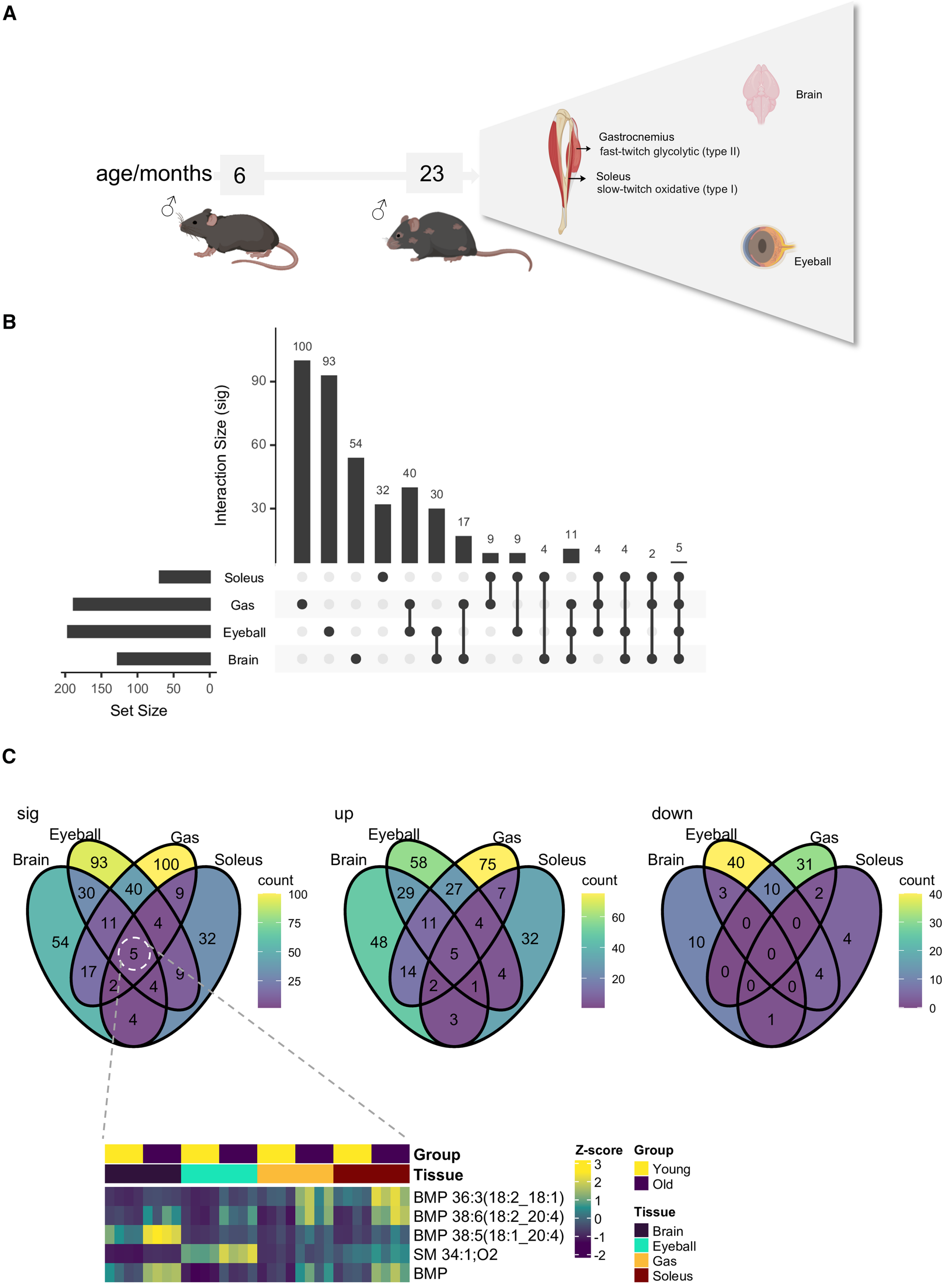

Figure 5. Multi-tiered methodology to interrogate senescence-associated remodeling in tissue organic acid profiles

(A) Senescence-associated changes in the lipid and organic acid profiles of the brain, eyeball (retina), and skeletal muscles of young (6-month-old, $n = 4$) and old (23-month-old, $n = 4$) C57BL/6N male mice were examined. The Gas muscle contains fast-twitch glycolytic (type II) muscle fibers, whereas the soleus comprises slow-twitch oxidative (type I) muscle fibers.

(legend continued on next page)

(DGs) containing esterified FA 18:1 in their structures (Figure 7A). Fractional distributions of esterified FA 18:1 were not significantly altered for PI 36:3(18:2_18:1) of both Gas and soleus muscles but marginally increased with age for DG 36:3 (18:2_18:1) in Gas muscles (Figure 7B), implying that excess bioavailability of FA 18:1 in aged Gas muscles was preferentially directed to DG over PI biosynthesis. Interestingly, the soleus muscle predominantly comprising oxidative myofibers also exhibited distinct accumulations in PEs containing esterified FA 20:4, including PE 38:5(18:1_20:4), PE O-38:6(O-18:2_20:4), and PE O-36:5(O-16:1_20:4) (Figure 7C), which might underlie the differential pace of aging observed between the oxidative and glycolytic muscles.

DISCUSSION

Aging is an intricate phenomenon characterized by progressive decline in physiological function, albeit the underlying principal causative mechanisms have remained largely elusive. Several theories of aging have been put forth, such as the free radical and mitochondrial theory of aging, which proposes reactive oxygen species (ROS)-induced mitochondrial damages as primary drivers of aging that subsequently lead to deteriorating mitochondrial function, propagating a vicious cycle of oxidative stress and mitochondrial damages.[34] Compromised mitochondrial function undermines β-oxidation, which can be reflected as changes in organic acid constitution, particularly FA profiles. In this work, we devised a multi-tiered workflow to examine fatty acyl partitioning in glycerolipids and validated our approach *in vitro* by using etomoxir-treated myotubes. Leveraging the three-arm workflow, we comprehensively captured age-associated alterations in endogenous organic acid profiles across multiple tissues of murine aging. These included the eyeball (retina) and the Gas muscle, which principally utilize glycolysis for energy production,[35] as well as the brain and the soleus muscle that are predominantly reliant on oxidative phosphorylation to meet their energy demand.[36] As tissues that primarily leverage oxidative phosphorylation have higher capacity to utilize FA fuels via β-oxidation compared with glycolytic tissues; it follows that glycolytic tissues may exhibit earlier aberrations in organic acid profiles as mitochondrial function deteriorates with aging.

In this study, we observed stark accumulation of esterified levels of OCFAs and diunsaturated FAs in the eyeball and Gas muscles of 23-month-old mice, which was evident neither in the brain nor the soleus. It was previously shown that OCFAs possess lower β-oxidizability relative to even-numbered FAs.[37] Furthermore, among diunsaturated FAs, FA 18:2 possessing two double bonds in their hydrocarbon chains was the least β-oxidized.[38] It appears that OCFAs and diunsaturated FAs, which are less preferentially oxidized and hence more easily assimilated into body fats, tend to accumulate in glycolytic tissues, such as the retina (eyeball) and the Gas muscle, when β-oxidation is compromised across aging. Consequently, an examination into the fractional distributions of esterified FAs using our established workflow uncovered that excess OCFAs were directed into TGs of aged tissues, which might account for the accretion of lipofuscin granules with aging. The lipid components of lipofuscin, also known as the age pigment that serves as a robust marker of human skeletal muscle and retina aging, are primarily represented by TGs.[39] Apart from organic acid perturbations conserved between the eyeball and the Gas muscle, our analyses also revealed distinct diminutions of VLC-PUFAs (C24–C32) in aged eyeball tissues. The concomitant reductions in both free and esterified VLC-PUFAs from our analyses suggest limiting bioavailability of VLC-PUFAs with age, which may be ascribed to either attenuated biosynthesis[40] or accelerated degradation due to ROS attacks.[41]

We also systematically compared the senescence-associated changes in organic acid signatures between the soleus and Gas muscles. The soleus comprises slow-twitch oxidative fibers with high mitochondria content, whereas the Gas muscle consists primarily of fast-twitch glycolytic fibers with very low mitochondria content.[42] We observed age-dependent increases in esterified FA 18:1, manifested as increases in PIs, PCs, PEs, and DGs containing oleic acid in their structures, which were conserved between the soleus and Gas muscles. Corroborating our findings, age-associated increases in PIs were previously reported in the skeletal muscles of mice,[28] as well as in the muscles of human volunteers,[28] which were proposed to be associated with accelerated aging.[43] PI biosynthesis requires the production of cytidine diphosphate diacylglycerol (CDP-DG) precursors—positioned at the branch point of phospholipid metabolism—that serve as intermediates to anionic phospholipids, distinguishing from an alternative pathway leading to neutral lipids and major phospholipids (PCs and PEs) via DGs.[44] Overexpression of CDP-DG synthase and PI synthase alone or in combination failed to enhance the rate of PI biosynthesis, which had led to the postulation that anionic phospholipid production was largely regulated by DG levels, instead of CDP-DG levels.[45] The amount of phosphatidic acids (PAs) available for CDP-DG synthase may be modulated by phosphatidic acid phosphorylase, which cleaves PAs to form DGs and shunt the bulk of the PAs to DGs over CDP-DG biosynthesis, thereby regulating cellular anionic phospholipid production.[45] In this study, we observed a preferential partitioning of oleic acid excess into DGs (over other phospholipids including PIs) in aged Gas muscles, which indicates that oleoyl-DG might be a central modulator of senescence-associated lipid signatures in skeletal muscle aging. Given that muscle-specific knockout of phosphatidic acid phosphorylase results in skeletal myopathy in mice,[46] we postulate that oleoyl-DGs might serve to buffer against pro-aging lipid modifications within skeletal muscles. Glycolytic skeletal muscles age faster than oxidative muscles in general. Glycolytic skeletal muscles

(B) UpSet plot illustrating the number of age-associated metabolite changes conserved across the four tissues investigated. The eyeball displayed the largest number of overlapping senescence-associated metabolites with the Gas muscles, both of which are glycolytic tissues.

(C) Venn diagrams summarizing the number of age-associated metabolites that were conserved across, or distinct to, the four tissues examined. BMPs were consistently elevated across all four tissues with age.

BMPs, bis(monoacylglycero)phosphates; up, upregulated with aging; down, downregulated with aging.

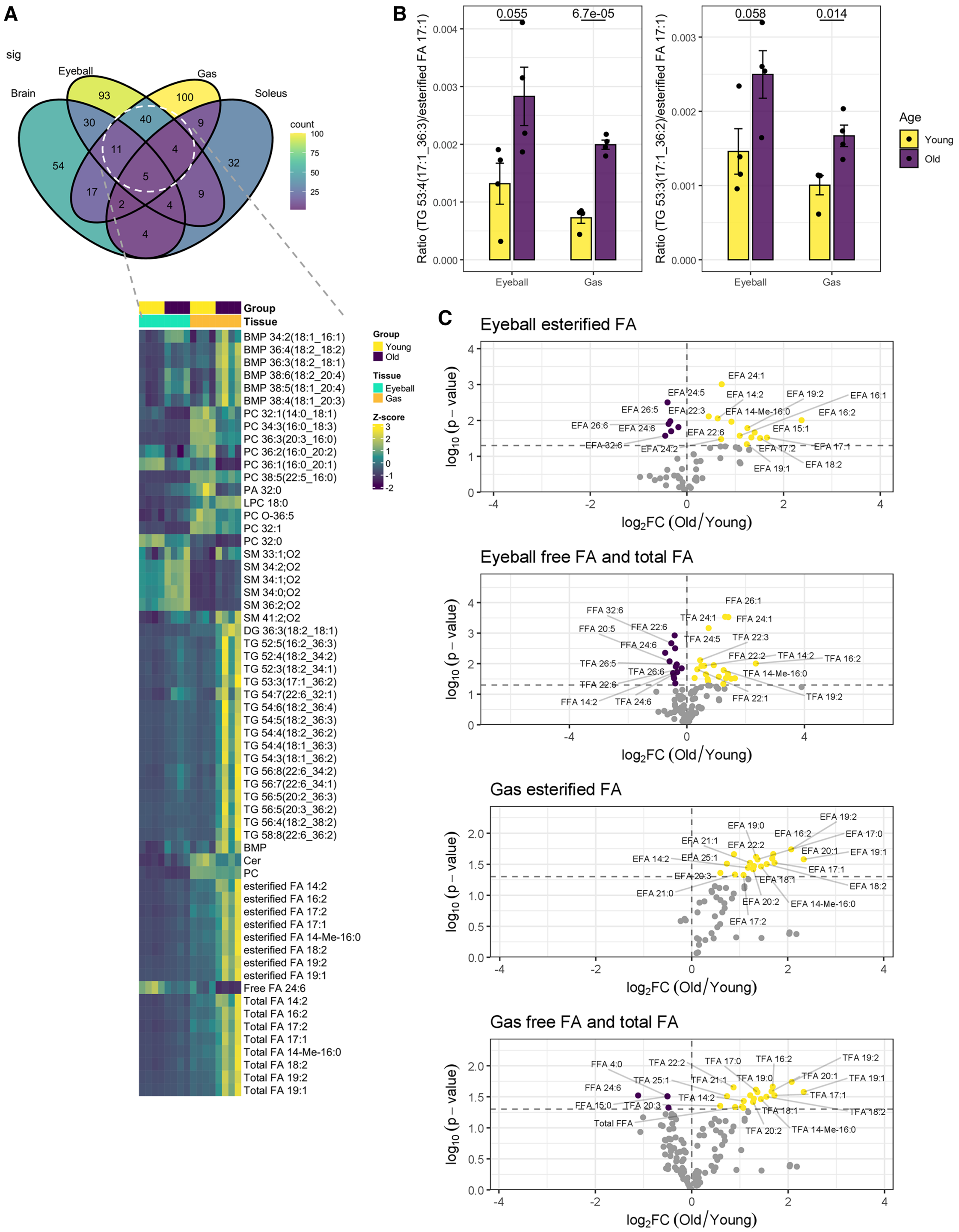

Figure 6. Age-associated changes in organic acid profiles of glycolytic tissues

(A) Heatmap illustrating age-associated metabolites conserved between the eyeball and Gas muscles, both of which rely principally on glycolysis for energy production.

(B) Bar plots displaying enhanced partitioning of esterified FA 17:1 toward TGs with aging in both eyeball and Gas muscles. *p* values from Student's *t* test are displayed. Error bars represent standard errors of the mean (SEM).

(C) Volcano plots illustrating FAs that were significantly altered in aged tissues relative to young tissues. Age-dependent increases are shown in yellow, whereas age-dependent reductions are shown in purple. Different endogenous forms of individual FAs, including esterified, free, and their total levels, were investigated. FFA: free fatty acid; EFA: esterified fatty acid; TFA: total fatty acid.

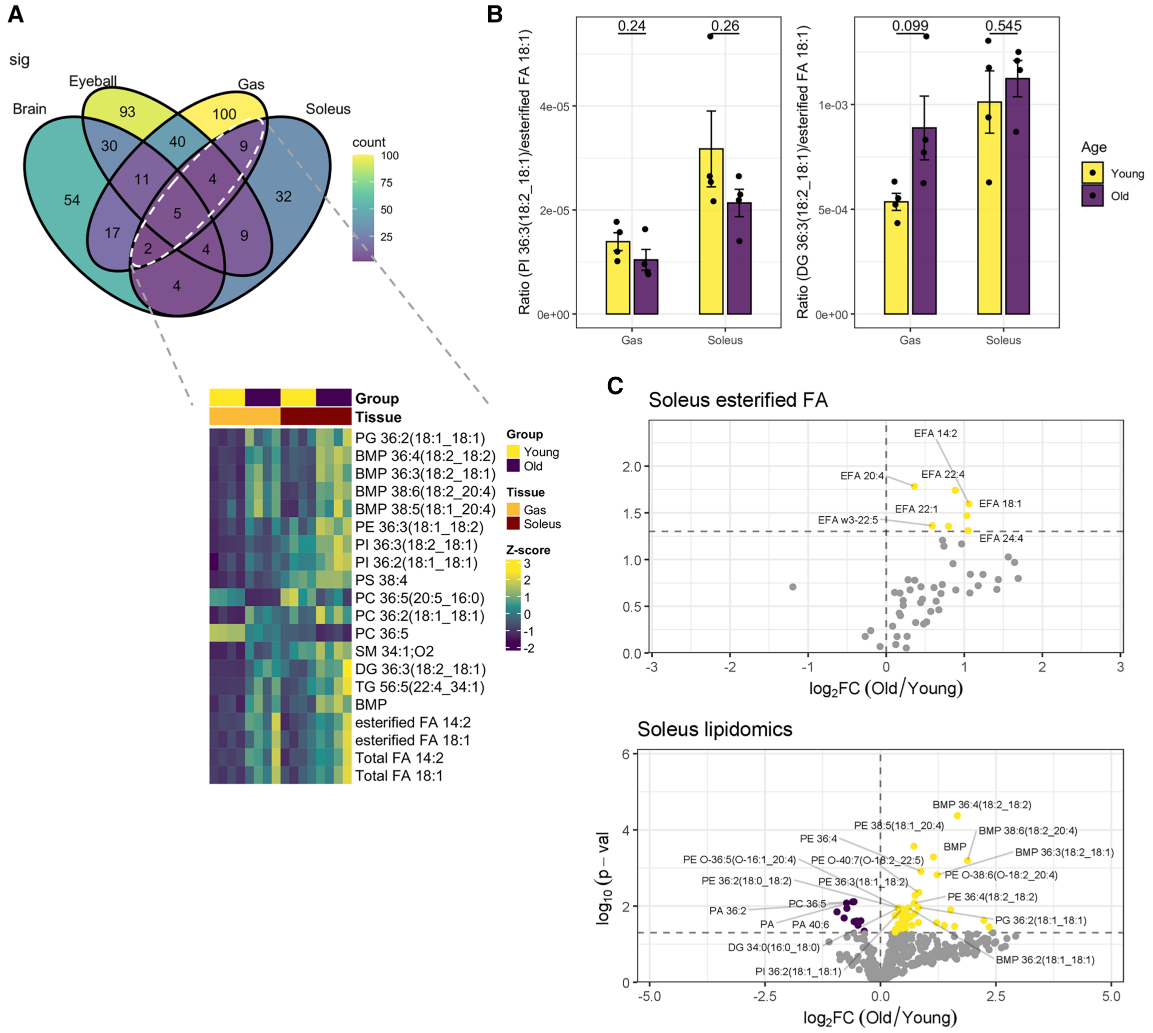

Figure 7. Age-associated changes in organic acid profiles of skeletal muscles

(A) Heatmap illustrating age-associated metabolites conserved between the Gas and soleus muscles, which depend on glycolysis and oxidative phosphorylation, respectively, for energy production. Both glycolytic and oxidative skeletal muscles displayed accretion in esterified FA18:1 with age.

(B) Bar plots displaying increased partitioning of esterified FA18:1 to DG 36:3(18:2_18:1), but not PI 36:3(18:2_18:1), in the Gas muscles. *p* values from Student's *t* test are displayed. Error bars represent standard errors of the mean (SEM).

(C) Volcano plots illustrating esterified FAs and individual lipids that were significantly altered in aged soleus muscles relative to young soleus muscles. Age-dependent increases are in yellow, whereas age-dependent reductions are in purple.

EFA: esterified fatty acid.

display greater myofiber atrophy than oxidative skeletal muscles across aging, and transcriptome profiles indicate that during aging, oxidative myofibers display elevated expression of antioxidant enzymes, while glycolytic myofibers exhibit higher expressions of genes related to muscle atrophy and inflammation.[47] We surmise that the observed accretions of esterified FA 20:4 in the aged soleus sequester arachidonic acids within lipid membranes and might serve to attenuate the arachidonic acid cascade,[48] which possibly underpins the slower pace of aging in the soleus compared to Gas muscles. Along this line, enhanced lipid membrane fluidity was reported to modulate muscle homeostasis during senescence via alleviation of endoplasmic reticulum stress, with higher fluidity (i.e., higher lipid hydrocarbon unsaturation) associated with young-like phenotype and greater muscle recovery.[49]

Limitations of the study

In this study, we innovated a multi-tiered workflow for a comprehensive evaluation of organic acid profiles within a single biological specimen, which entails complete characterization and quantification of polar carboxylic acids constituting the TCA cycle, as well as C2–C34 FAs in varying endogenous forms i.e., free or esterified, to different lipid classes/species. Our three-tiered workflow allows evaluation of the fractional distributions of esterified fatty acyls across different lipid classes/species, which confers useful biological insights into fatty

acyl partitioning and cellular energy metabolism. Our methodology has limitations. A direct method for quantifying free FAs or those released by saponification—without derivatization—offers clear advantages in terms of speed and cost. However, derivatization with 3-NPH significantly enhanced LC-MS analysis by improving ionization efficiency, chromatographic separation, linearity, and LODs. Derivatization with 3-NPH also mitigated the chromatographic incompatibility resulting from the broad polarity spectrum of organic acids. In order to accommodate the wide polarity spectrum spanning SCFAs, MCFAs, LCFAs, and VLCFAs within a single injection, our choice of resuspension solvent could reduce the solubility and sensitivity of detection for SCFAs relative to the longer-chain FAs. Nonetheless, our methodology offers a comprehensive screen of endogenous organic acid profiles based on limiting amounts of biological specimens, rendering a thorough evaluation of energy metabolism defined by organic acid homeostasis.

RESOURCE AVAILABILITY

Lead contact

Further information and requests for resources should be directed to and will be fulfilled by the lead contact, Sin Man Lam (smlam@lipidall.com).

Materials availability

This study did not generate new unique reagents.

Data and code availability

- Data have been deposited at Zenodo and are publicly available as of the date of publication at https://doi.org/10.5281/zenodo.18797611.
- This paper does not report original code.
- Any additional information required to reanalyze the data reported in this work paper is available from the lead contact upon request.

ACKNOWLEDGMENTS

This work was supported by the Major Project of Guangzhou National Laboratory (GZNL2024A03013) to G.S. and the National Key R&D Program of China (2022YFA0806001) to S.M.L.

AUTHOR CONTRIBUTIONS

Conceptualization, S.M.L. and G.S.; methodology, S.M.L., Z.Z, and T.L.; formal analysis, Z.Z., C.C., Y.R., T.L., and B.L.; investigation, Z.Z., C.C., Y.R., T.L., M.C., and L.M.; writing – original draft, S.M.L.; writing – review & editing, Z.Z., C.C., B.L., and G.S.; resources, S.M.L. and G.S.; funding acquisition, G.S. and S.M.L.; supervision, S.M.L. and G.S.

DECLARATION OF INTERESTS

Z.Z., Y.R., T.L., B.L., and S.M.L. are employees of LipidALL Technologies.

STAR★METHODS

Detailed methods are provided in the online version of this paper and include the following:

- KEY RESOURCES TABLE
- EXPERIMENTAL MODEL AND STUDY PARTICIPANT DETAILS
  - Animals
  - Mouse C2C12 myoblast culture
- METHOD DETAILS
  - Chemical reagents
  - Treatment of C2C12 myotube with etomoxir
  - Extraction of lipids (including FAs) and organic acids
  - Lipid saponification to derive total FAs (sum of free and esterified FAs)
  - Chemical derivatisation of carboxylic acids with 3-NPH
  - Organic acid analyses
  - Lipidomics analyses
- QUANTIFICATION AND STATISTICAL ANALYSIS

SUPPLEMENTAL INFORMATION

Supplemental information can be found online at https://doi.org/10.1016/j.crmeth.2026.101413.

Received: May 21, 2025
Revised: January 1, 2026
Accepted: March 24, 2026
Published: April 21, 2026

REFERENCES

1. Guo, K., and Li, L. (2010). High-performance isotope labeling for profiling carboxylic acid-containing metabolites in biofluids by mass spectrometry. Anal. Chem. *82*, 8789–8793. https://doi.org/10.1021/ac102146g.
2. Lam, S.M., Zhou, T., Li, J., Zhang, S., Chua, G.H., Li, B., and Shui, G. (2020). A robust, integrated platform for comprehensive analyses of acyl-coenzyme As and acyl-carnitines revealed chain length-dependent disparity in fatty acyl metabolic fates across Drosophila development. Sci. Bull. *65*, 1840–1848. https://doi.org/10.1016/j.scib.2020.07.023.
3. Lam, S.M., Wang, Z., Li, J., Huang, X., and Shui, G. (2017). Sequestration of polyunsaturated fatty acids in membrane phospholipids of Caenorhabditis elegans dauer larva attenuates eicosanoid biosynthesis for prolonged survival. Redox Biol. *12*, 967–977. https://doi.org/10.1016/j.redox.2017.05.002.
4. Lam, S.M., Chua, G.H., Li, X.J., Su, B., and Shui, G. (2016). Biological relevance of fatty acyl heterogeneity to the neural membrane dynamics of rhesus macaques during normative aging. Oncotarget *7*, 55970–55989. https://doi.org/10.18632/oncotarget.11190.
5. Parisi, L.R., Li, N., and Atilla-Gokcumen, G.E. (2017). Very Long Chain Fatty Acids Are Functionally Involved in Necroptosis. Cell Chem. Biol. *24*, 1445–1454.e8. https://doi.org/10.1016/j.chembiol.2017.08.026.
6. McMahon, A., Jackson, S.N., Woods, A.S., and Kedzierski, W. (2007). A Stargardt disease-3 mutation in the mouse Elovl4 gene causes retinal deficiency of C32-C36 acyl phosphatidylcholines. FEBS Lett. *581*, 5459–5463. https://doi.org/10.1016/j.febslet.2007.10.050.
7. Robinson, B.S., Johnson, D.W., and Poulos, A. (1990). Unique molecular species of phosphatidylcholine containing very-long-chain (C24-C38) polyenoic fatty acids in rat brain. Biochem. J. *265*, 763–767. https://doi.org/10.1042/bj2650763.
8. Khaw, K.T., Friesen, M.D., Riboli, E., Luben, R., and Wareham, N. (2012). Plasma phospholipid fatty acid concentration and incident coronary heart disease in men and women: the EPIC-Norfolk prospective study. PLoS Med. *9*, e1001255. https://doi.org/10.1371/journal.pmed.1001255.
9. Hodge, A.M., English, D.R., O'Dea, K., Sinclair, A.J., Makrides, M., Gibson, R.A., and Giles, G.G. (2007). Plasma phospholipid and dietary fatty acids as predictors of type 2 diabetes: interpreting the role of linoleic acid. Am. J. Clin. Nutr. *86*, 189–197. https://doi.org/10.1093/ajcn/86.1.189.
10. Zhang, W., Guo, J., Miao, G., Chen, J., Xu, Y., Lai, P., Zhang, L., Han, Y., Lam, S.M., Shui, G., et al. (2025). Fat-1 Ameliorates Metabolic Dysfunction-Associated Fatty Liver Disease and Atherosclerosis through Promoting the Nuclear Localization of PPARα in Hamsters. Research *8*, 0577. https://doi.org/10.34133/research.0577.
11. Huynh, K., Lim, W.L.F., Giles, C., Jayawardana, K.S., Salim, A., Mellett, N.A., Smith, A.A.T., Olshansky, G., Drew, B.G., Chatterjee, P., et al.

CellPress
OPEN ACCESS

(2020). Concordant peripheral lipidome signatures in two large clinical studies of Alzheimer's disease. Nat. Commun. *11*, 5698. https://doi.org/10.1038/s41467-020-19473-7.

12. Wan, L.Y., Lam, S.M., Huang, H.H., Cao, W.J., Cao, X.Y., Li, X.M., Zhang, L.P., Gao, J.M., Zhang, C., Fan, X., et al. (2025). Multi-omics dissection of metabolic dysregulation associated with immune recovery in people living with HIV-1. J. Transl. Med. *23*, 143. https://doi.org/10.1186/s12967-025-06168-0.
13. Lam, S.M., Wang, Z., Song, J.W., Shi, Y., Liu, W.Y., Wan, L.Y., Duan, K., Chua, G.H., Zhou, Y., Wang, G., et al. (2025). Non-invasive lipid panel of MASLD fibrosis transition underscores the role of lipoprotein sulfatides in hepatic immunomodulation. Cell Metab. *37*, 69–86.e7. https://doi.org/10.1016/j.cmet.2024.09.009.
14. Zhang, L., Zhao, J., Lam, S.M., Chen, L., Gao, Y., Wang, W., Xu, Y., Tan, T., Yu, H., Zhang, M., et al. (2024). Low-input lipidomics reveals lipid metabolism remodelling during early mammalian embryo development. Nat. Cell Biol. *26*, 278–293. https://doi.org/10.1038/s41556-023-01341-3.
15. Kang, J.X., and Wang, J. (2005). A simplified method for analysis of polyunsaturated fatty acids. BMC Biochem. *6*, 5. https://doi.org/10.1186/1471-2091-6-5.
16. Li, X., and Franke, A.A. (2011). Improved LC-MS method for the determination of fatty acids in red blood cells by LC-orbitrap MS. Anal. Chem. *83*, 3192–3198. https://doi.org/10.1021/ac103093w.
17. Yang, W.C., Adamec, J., and Regnier, F.E. (2007). Enhancement of the LC/MS analysis of fatty acids through derivatization and stable isotope coding. Anal. Chem. *79*, 5150–5157. https://doi.org/10.1021/ac070311t.
18. Jiang, R., Jiao, Y., Zhang, P., Liu, Y., Wang, X., Huang, Y., Zhang, Z., and Xu, F. (2017). Twin Derivatization Strategy for High-Coverage Quantification of Free Fatty Acids by Liquid Chromatography-Tandem Mass Spectrometry. Anal. Chem. *89*, 12223–12230. https://doi.org/10.1021/acs.analchem.7b03020.
19. Ribeiro, W., Vinolo, M., Calixto, L., and Ferreira, C. (2018). Use of Gas Chromatography to Quantify Short Chain Fatty Acids in the Serum, Colonic Luminal Content and Feces of mice. Bio. Protoc. *8*, e3089. https://doi.org/10.21769/BioProtoc.3089.
20. Han, J., Lin, K., Sequeira, C., and Borchers, C.H. (2015). An isotope-labeled chemical derivatization method for the quantitation of short-chain fatty acids in human feces by liquid chromatography-tandem mass spectrometry. Anal. Chim. Acta *854*, 86–94. https://doi.org/10.1016/j.aca.2014.11.015.
21. Han, J., Gagnon, S., Eckle, T., and Borchers, C.H. (2013). Metabolomic analysis of key central carbon metabolism carboxylic acids as their 3-nitrophenylhydrazones by UPLC/ESI-MS. Electrophoresis *34*, 2891–2900. https://doi.org/10.1002/elps.201200601.
22. Li, B., Li, L., Li, M., Lam, S.M., Wang, G., Wu, Y., Zhang, H., Niu, C., Zhang, X., Liu, X., et al. (2019). Microbiota Depletion Impairs Thermogenesis of Brown Adipose Tissue and Browning of White Adipose Tissue. Cell Rep. *26*, 2720–2737.e5. https://doi.org/10.1016/j.celrep.2019.02.015.
23. Wang, W., Li, S., Li, Z., Zhang, J., Fan, K., Tan, G., Ai, G., Lam, S.M., Shui, G., Yang, Z., et al. (2020). Harnessing the intracellular triacylglycerols for titer improvement of polyketides in Streptomyces. Nat. Biotechnol. *38*, 76–83. https://doi.org/10.1038/s41587-019-0335-4.
24. Pfeuffer, M., and Jaudszus, A. (2016). Pentadecanoic and Heptadecanoic Acids: Multifaceted Odd-Chain Fatty Acids. Adv. Nutr. *7*, 730–734. https://doi.org/10.3945/an.115.011387.
25. Jackson, S.J., Andrews, N., Ball, D., Bellantuono, I., Gray, J., Hachoumi, L., Holmes, A., Latcham, J., Petrie, A., Potter, P., et al. (2017). Does age matter? The impact of rodent age on study outcomes. Lab. Anim. *51*, 160–169. https://doi.org/10.1177/0023677216653984.
26. Cabrero, A., Alegret, M., Sánchez, R., Adzet, T., Laguna, J.C., and Vázquez, M. (2001). Uncoupling protein-3 mRNA up-regulation in C2C12 myotubes after etomoxir treatment. Biochim. Biophys. Acta *1532*, 195–202. https://doi.org/10.1016/s1388-1981(01)00131-7.
27. Fujiwara, K., Asai, H., Toyama, H., Kunita, K., Yaguchi, C., Kiyota, N., Tomita, H., and Jacobs, J.V. (2010). Changes in muscle thickness of gastrocnemius and soleus associated with age and sex. Aging Clin. Exp. Res. *22*, 24–30. https://doi.org/10.1007/bf03324811.
28. Janssens, G.E., Molenaars, M., Herzog, K., Grevendonk, L., Remie, C.M.E., Vervaart, M.A.T., Elfrink, H.L., Wever, E.J.M., Schomakers, B.V., Denis, S.W., et al. (2024). A conserved complex lipid signature marks human muscle aging and responds to short-term exercise. Nat. Aging *4*, 681–693. https://doi.org/10.1038/s43587-024-00595-2.
29. Wang, D., Xu, P., and Mesaros, C. (2021). Analytical considerations for reducing the matrix effect for the sphingolipidome quantification in whole blood. Bioanalysis *13*, 1037–1049. https://doi.org/10.4155/bio-2021-0098.
30. Liu, A., Chang, J., Lin, Y., Shen, Z., and Bernstein, P.S. (2010). Long-chain and very long-chain polyunsaturated fatty acids in ocular aging and age-related macular degeneration. J. Lipid Res. *51*, 3217–3229. https://doi.org/10.1194/jlr.M007518.
31. Dornstauder, B., Suh, M., Kuny, S., Gaillard, F., Macdonald, I.M., Clandinin, M.T., and Sauvé, Y. (2012). Dietary docosahexaenoic acid supplementation prevents age-related functional losses and A2E accumulation in the retina. Investig. Ophthalmol. Vis. Sci. *53*, 2256–2265. https://doi.org/10.1167/iovs.11-8569.
32. Prokopiou, E., Kolovos, P., Georgiou, C., Kalogerou, M., Potamiti, L., Sokratous, K., Kyriacou, K., and Georgiou, T. (2019). Omega-3 fatty acids supplementation protects the retina from age-associated degeneration in aged C57BL/6J mice. BMJ Open Ophthalmol. *4*, e000326. https://doi.org/10.1136/bmjophth-2019-000326.
33. Rotstein, N.P., Ilincheta de Boschero, M.G., Giusto, N.M., and Aveldaño, M.I. (1987). Effects of aging on the composition and metabolism of docosahexaenoate-containing lipids of retina. Lipids *22*, 253–260. https://doi.org/10.1007/bf02533988.
34. Cui, H., Kong, Y., and Zhang, H. (2012). Oxidative stress, mitochondrial dysfunction, and aging. J. Signal Transduct. *2012*, 1–13. https://doi.org/10.1155/2012/646354.
35. Wang, L., Törnquist, P., and Bill, A. (1997). Glucose metabolism in pig outer retina in light and darkness. Acta Physiol. Scand. *160*, 75–81. https://doi.org/10.1046/j.1365-201X.1997.00030.x.
36. Hall, C.N., Klein-Flügge, M.C., Howarth, C., and Attwell, D. (2012). Oxidative phosphorylation, not glycolysis, powers presynaptic and postsynaptic mechanisms underlying brain information processing. J. Neurosci. *32*, 8940–8951. https://doi.org/10.1523/jneurosci.0026-12.2012.
37. Shibata, R., Gotoh, N., Kubo, A., Kanda, J., Nagai, T., Mizobe, H., Yoshinaga, K., Kojima, K., Watanabe, H., and Wada, S. (2012). Comparison of catabolism rate of fatty acids to carbon dioxide in mice. Eur. J. Lipid Sci. Technol. *114*, 1340–1344. https://doi.org/10.1002/ejlt.201200164.
38. DeLany, J.P., Windhauser, M.M., Champagne, C.M., and Bray, G.A. (2000). Differential oxidation of individual dietary fatty acids in humans. Am. J. Clin. Nutr. *72*, 905–911. https://doi.org/10.1093/ajcn/72.4.905.
39. Terman, A., and Brunk, U.T. (2004). Lipofuscin. Int. J. Biochem. Cell Biol. *36*, 1400–1404. https://doi.org/10.1016/j.biocel.2003.08.009.
40. Chen, D., Chao, D.L., Rocha, L., Kolar, M., Nguyen Huu, V.A., Krawczyk, M., Dasyani, M., Wang, T., Jafari, M., Jabari, M., et al. (2020). The lipid elongation enzyme ELOVL2 is a molecular regulator of aging in the retina. Aging Cell *19*, e13100. https://doi.org/10.1111/acel.13100.
41. Yin, H., and Porter, N.A. (2005). New insights regarding the autoxidation of polyunsaturated fatty acids. Antioxid. Redox Signal. *7*, 170–184. https://doi.org/10.1089/ars.2005.7.170.
42. Tucci, S., Mingirulli, N., Wehbe, Z., Dumit, V.I., Kirschner, J., and Spiekerkoetter, U. (2018). Mitochondrial fatty acid biosynthesis and muscle fiber plasticity in very long-chain acyl-CoA dehydrogenase-deficient mice. FEBS Lett. *592*, 219–232. https://doi.org/10.1002/1873-3468.12940.

43. Liu, D., Aziz, N.A., Landstra, E.N., and Breteler, M.M.B. (2023). The lipidomic correlates of epigenetic aging across the adult lifespan: A population-based study. Aging Cell *22*, e13934. https://doi.org/10.1111/acel.13934.

44. Kent, C. (1995). Eukaryotic phospholipid biosynthesis. Annu. Rev. Biochem. *64*, 315–343. https://doi.org/10.1146/annurev.bi.64.070195.001531.

45. Lykidis, A., Jackson, P.D., Rock, C.O., and Jackowski, S. (1997). The role of CDP-diacylglycerol synthetase and phosphatidylinositol synthase activity levels in the regulation of cellular phosphatidylinositol content. J. Biol. Chem. *272*, 33402–33409. https://doi.org/10.1074/jbc.272.52.33402.

46. Schweitzer, G.G., Collier, S.L., Chen, Z., McCommis, K.S., Pittman, S.K., Yoshino, J., Matkovich, S.J., Hsu, F.F., Chrast, R., Eaton, J.M., et al. (2019). Loss of lipin 1-mediated phosphatidic acid phosphohydrolase activity in muscle leads to skeletal myopathy in mice. Faseb j *33*, 652–667. https://doi.org/10.1096/fj.201800361R.

47. Zhang, F.M., Wu, H.F., Wang, K.F., Yu, D.Y., Zhang, X.Z., Ren, Q., Chen, W.Z., Lin, F., Yu, Z., and Zhuang, C.L. (2024). Transcriptome profiling of fast/glycolytic and slow/oxidative muscle fibers in aging and obesity. Cell Death Dis. *15*, 459. https://doi.org/10.1038/s41419-024-06851-y.

48. Korotkova, M., and Lundberg, I.E. (2014). The skeletal muscle arachidonic acid cascade in health and inflammatory disease. Nat. Rev. Rheumatol. *10*, 295–303. https://doi.org/10.1038/nrrheum.2014.2.

49. Lee, S.M., Lee, S.H., Jung, Y., Lee, Y., Yoon, J.H., Choi, J.Y., Hwang, C.Y., Son, Y.H., Park, S.S., Hwang, G.S., et al. (2020). FABP3-mediated membrane lipid saturation alters fluidity and induces ER stress in skeletal muscle with aging. Nat. Commun. *11*, 5661. https://doi.org/10.1038/s41467-020-19501-6.

50. Miao, H., Li, B.W., Wang, Z.H., Mu, J.M., Tian, Y.N., Jiang, B.H., Zhang, S.H., Gong, X., Shui, G.H., and Lam, S.M. (2022). Lipidome Atlas of the Developing Heart Uncovers Dynamic Membrane Lipid Attributes Underlying Cardiac Structural and Metabolic Maturation. Research *2022*, 0006. https://doi.org/10.34133/research.0006.

STAR★METHODS

KEY RESOURCES TABLE

REAGENT or RESOURCE	SOURCE	IDENTIFIER
Chemicals, peptides, and recombinant proteins		
chloroform	CNW	CAEQ-4-011024-4000
methanol	Fisher	A452-4
acetonitrile	Fisher	A998-4
isopropanol	Fisher	A451-4
butylated hydroxytoluene(BHT)	Sigma-Aldrich	B1378
potassium hydroxide(KOH)	SCR	10017018
hydrochloric acid(HCl)	SCR	10011018
n-hexane	CNW	CAEQ-4-011516-4000
N-(3-Dimethylaminopropyl)-N′-ethylcarbodiimide hydrochloride(EDC.HCl)	Sigma-Aldrich	E6383
pyridine	Sigma-Aldrich	360567
formic acid	Sigma-Aldrich	F0507
3-nitrophenylhydrazine hydrochloride(3-NPH.HCl)	Sigma-Aldrich	N21804
ammonium acetate	Sigma-Aldrich	A7330
ammonium hydroxide	Sigma-Aldrich	5002
d_{31}-FA-C16:0	Sigma-Aldrich	366897
d_8-FA-C20:4	Cayman Chemicals	390010
butyric-2,2-d_2-acid	CDN isotopes	ND 0976
octanoic acid-1-^{13} C	Sigma-Aldrich	296457
2,2,3,3-d_4-succinic acid	CIL	DLM 584
d_4-fumaric acid	CIL	DLM 7654
2,2,4,4-d_4-citric acid	CIL	DLM-3487
2,3,3-d_3-malic acid	CIL	DLM 9045
d_3-pyruvic acid	CIL	DLM 6068
d_3-lactic acid	Sigma-Aldrich	616567
d_9-PC 32:0(16:0/16:0)	Avanti	860352P
PE 17:0/17:0	Avanti	830756P
d_{31}-PS	Avanti	860403P
PA17:0/17:0	Avanti	830856P
PG14:0/14:0	Avanti	840445P
CL14:0/14:0/14:0/14:0	Avanti	750332P
BMP 14:0/14:0	Avanti	857131P
SL d18:1/12:0	Avanti	860573P
LPC 17:0	Avanti	855676P
LPI 17:1	Avanti	850103P
LPA 17:0	Avanti	857127P
LPS17:1	Avanti	110724
Cer d18:1/17:0	Avanti	860517P
SM d18:1/12:0	Avanti	860583P
S1P d17:1	Avanti	860641P
Sph d17:1	Avanti	860640P
GluCer d18:1/8:0	Avanti	860540P
LacCer d18:1/8:0	Avanti	860541P
Gb3 d18:1/17:0	Matreya LLC	1523

(Continued on next page)

Continued		
REAGENT or RESOURCE	SOURCE	IDENTIFIER
d_3-16:0 carnitine	Cambridge isotopes	DLM 1263
DAG 18:1/18:1-d_5	Avanti	110581
diC8-PI	Echelon	P-0008
GM3 d18:1/18:0-d_3	Matreya LLC	2052
d_6-CE18:0	CDN isotopes	D5823
TAG$(16:0)_3$-d_5	CDN isotopes	D5815
Etomoxir	MCE	HY-50202
water	Merck Millipore	Milli-Q system
Deposited data		
Source data for individual figures and tables	This paper	Zenodo, https://doi.org/10.5281/zenodo.18797611
Experimental models: Cell lines		
Cell: C2C12	ATCC	RRID: CVCL_0188
Experimental models: Organisms/strains		
Mouse: C57BL/6NCrl	Beijing Vital River Laboratory Animal Technologies Co. Ltd.	RRID: IMSR_CRL:027

EXPERIMENTAL MODEL AND STUDY PARTICIPANT DETAILS

Animals

C57BL/6N male mice (*n* = 8) were purchased from Beijing Vital River Laboratory Animal Technologies Co. Ltd. The analysis was restricted to male mice in order to minimize the effects of hormonal variability on age-associated lipid metabolism. The estrous cycle in females introduces cyclical hormonal fluctuations that directly influence lipid metabolism and henceforth tissue-specific organic acid profiles. These fluctuations increase intra-group variability and could obscure aging-related effects unless extremely large sample sizes are used. The use of male mice thus allows for investigation of aging-associated lipid changes with less confounders.

The mice were kept in a temperature-controlled environment set at 22 ± 1°C with 12-h light and dark cycle and *ad libitum* access to food and water. The mice were individually housed and fed with a standard AIN-93M diet. Mice were anesthetized by intraperitoneal injection with avertin (20 μL/g body mass), following which tissues were collected from young (6-month-old) male mice (*n* = 4) and old (23-month-old) male mice (*n* = 4). All animal handling procedures were approved by the Animal Care and Use Committee at the Institute of Genetics and Developmental Biology, Chinese Academy of Sciences.

Mouse C2C12 myoblast culture

Mouse C2C12 myoblasts (ATCC) were maintained in Dulbecco's modified Eagle's medium (DMEM, Gibco) supplemented with 10% fetal bovine serum (FBS, Gibco) and 1% penicillin-streptomycin solution (p/s, Gibco). Upon reaching approximately 80% confluence, the growth medium was replaced with differentiation medium consisting of DMEM containing 2% horse serum (HS, Gibco) and 1% penicillin-streptomycin Solution. After 4–5 days of further culture, during which the cells fused to form myotubes, the myotubes were switched to serum-free medium prior to drug treatment.

METHOD DETAILS

Chemical reagents

HPLC grade chloroform (CNW CAEQ-4-011024-4000), methanol (Fisher A452-4), acetonitrile (Fisher A998-4), isopropanol (Fisher A451-4), butylated hydroxytoluene (BHT, Sigma-Aldrich B1378), potassium hydroxide (KOH, SCR 10017018), hydrochloric acid (HCl, SCR 10011018), n-hexane (CNW CAEQ-4-011516-4000), N-(3-Dimethylaminopropyl)-N′-ethylcarbodiimide hydrochloride (EDC.HCl, Sigma-Aldrich E6383), pyridine (Sigma-Aldrich 360567), formic acid (Sigma-Aldrich F0507), 3-nitrophenylhydrazine hydrochloride (3-NPH.HCl, Sigma-Aldrich N21804), ammonium acetate (Sigma-Aldrich A7330), ammonium hydroxide (Sigma-Aldrich 05002), d31-FA-C16:0 (Sigma-Aldrich 366897), d8-FA-C20:4 (Cayman Chemicals 390010), butyric-2,2-d2-acid (CDN isotopes ND 0976), octanoic acid-1-13C (Sigma-Aldrich 296457), 2,2,3,3-d4-succinic acid (CIL DLM 584), d4-fumaric acid (CIL DLM 7654), 2,2,4,4-d4-citric acid (CIL DLM 3487), 2,3,3-d3-malic acid (CIL DLM 9045), d3-pyruvic acid (CIL DLM 6068), d3-lactic acid (Sigma-Aldrich 616567), d9-PC 32:0(16:0/16:0) (Avanti 860352P), PE 17:0/17:0 (Avanti 830756P), d31-PS (Avanti 860403P), PA17:0/17:0 (Avanti 830856P), PG14:0/14:0 (Avanti 840445P), CL14:0/14:0/14:0/14:0 (Avanti 750332P), BMP 14:0/14:0 (Avanti 857131P), SL d18:1/12:0 (Avanti 860573P), LPC 17:0 (Avanti 855676P), LPI 17:1 (Avanti 850103P), LPA 17:0 (Avanti 857127P), LPS17:1 (Avanti 110724), Cer d18:1/17:0 (Avanti 860517P), SM d18:1/12:0 (Avanti 860583P), S1P d17:1 (Avanti 860641P), Sph

d17:1 (Avanti 860640P), GluCer d18:1/8:0 (Avanti 860540P), LacCer d18:1/8:0 (Avanti 860541P), Gb3 d18:1/17:0 (Matreya LLC 1523), d3-16:0 carnitine (Cambridge isotopes DLM 1263), DAG 18:1/18:1-d5 (Avanti 110581), diC8-PI (Echelon P-0008), GM3 d18:1/18:0-d3 (Matreya LLC 2052), d6-CE18:0 (CDN isotopes D5823), TAG(16:0)3-d5 (CDN isotopes D5815) and Etomoxir (MCE HY-50202) were used. LC-MS grade water was dispensed from a Milli-Q system (Merck Millipore, Burlington, MA).

Treatment of C2C12 myotube with etomoxir

Prior to drug treatment, the myotubes were switched to serum-free medium and assigned to three treatment groups: control group, 40 μM etomoxir-treated group, and 80 μM etomoxir-treated group, each with six biological replicates. After 24 h of treatment, the medium was aspirated and the myotubes were gently washed with phosphate-buffered saline (PBS). The cells were then detached by incubation with trypsin for 3–5 min at 37°C. Trypsinization was terminated by adding DMEM supplemented with 10% FBS. The cell suspension was collected into 1.5 mL microcentrifuge tubes and centrifuged at 800 × g for 4 min at 4°C. The supernatant was discarded, and the cell pellet was washed once with PBS by gentle pipetting. Following a second centrifugation under the same conditions, all PBS was completely aspirated, leaving only the cell pellet, which was subsequently stored at −80°C until further analyses. Lipid and organic acid extractions were carried out as elaborated for tissue samples.

Extraction of lipids (including FAs) and organic acids

Lipid extraction was performed on cultured myotubes and mice tissues (≈30 mg) including the brain, eyeball, skeletal muscles (the gastrocnemius and soleus) using a modified Bligh and Dyer's method.[50] In brief, 50 μL of internal standard cocktail for SCFA and polar carboxylic acid quantification were added at lipid extraction, which contained octanoic acid-1-^{13} C (1 μg/mL, Sigma-Aldrich 296457), butyric-2,2-d_2-acid (10 μg/mL, CDN isotopes ND 0976), 2,2,3,3-d_4-succinic acid (5 μg/mL, CIL DLM 584), d_4-fumaric acid (10 μg/mL, CIL DLM 7654), 2,2,4,4-d_4-citric acid (20 μg/mL, CIL DLM-3487), 2,3,3-d_3-malic acid (5 μg/mL, CIL DLM 9045), d_3-pyruvic acid (10 μg/mL, CIL DLM 6068) and d_3-lactic acid (20 μg/mL, Sigma-Aldrich 616567) dissolved in acetonitrile: MilliQ-H_2O (1:1, v/v). Following this, 900 μL of extraction solvent comprising chloroform: methanol: MilliQ-H_2O (3:6:1 v/v) containing 0.01% (w/v) of butylated hydroxytoluene (BHT) was added. Tissues were homogenized on an automated bead ruptor (programmed at 6 m/s for 5 s at 4°C, 4 cycles, with an interval of 3s), then incubated at 1500 rpm for 30 min at 4°C. At the end of the incubation, 350 μL of MilliQ-H_2O and 300 μL of chloroform were added to the samples to induce phase separation. The samples were then centrifuged at 12,000 rpm at 4°C for 5 min and the lower organic phase containing lipids were transferred into a clean 1.5 mL Eppendorf safe-lock tube. Lipid extraction was repeated once by adding 500 μL of chloroform to the remaining aqueous phase. The lower organic phase from both rounds was pooled together and dried in the SpeedVac under organic mode. The dried extract was resuspended in chloroform: methanol (1:1, v/v), split into three portions, each portion designated for subsequent processing under Branches A, B and C of the multi-tiered workflow, respectively (Figure 1), then dried again using the SpeedVac in the organic mode and stored at −80°C until further analysis.

Lipid saponification to derive total FAs (sum of free and esterified FAs)

Alkaline hydrolysis was performed on the portion of lipid extract designated for total FA analysis (Figure 1, branch B). In brief, internal standard cocktail for FA quantification comprising d_{31}-FA-C16:0 (5 μg/mL, Sigma-Aldrich 366897) and d_8-FA-C20:4 (5 μg/mL, Cayman Chemicals 390010) was added to the dried lipid extract, followed by 500 μL of 10% KOH. The samples were incubated at 80°C for 2 h. After incubation, the samples were allowed to cool to room temperature, then 133 μL of 6N HCl was added to neutralize the KOH in the reaction mixture. The saponified lipid extract was dried in a SpeedVac under OH mode. Then, 1000 μL of n-hexane: isopropanol (3:2, v/v) was added, and samples were incubated at 1500 rpm for 30 min at 4°C. The samples were centrifuged at 12,000 rpm for 5 min at 4°C post incubation. Clean supernatant (980 μL) was transferred to a new 2 mL Eppendorf safe-lock tube. A second round of lipid extraction was performed by adding 1000 μL of n-hexane: isopropanol (3:2, v/v) to the remaining pellet, which was completely dislodged by vortexing. The two rounds of dried extracts containing saponified FAs (or total FAs) were dried in the SpeedVac under OH mode, resuspended in 180 mM of N-(3-Dimethylaminopropyl)-N′-ethylcarbodiimide hydrochloride (EDC.HCl, Sigma-Aldrich E6383) containing 7.5% (v/v) of pyridine (Sigma-Aldrich 360567) dissolved in methanol: isopropanol (3:7, v/v), then proceeded with chemical derivatisation using 3-NPH.

Chemical derivatisation of carboxylic acids with 3-NPH

For the portion of lipid extract designated for FFA analysis (Figure 1, branch C), an internal standard cocktail (50 μL) for FA quantification comprising d_{31}-FA-C16:0 (5 μg/mL, Sigma-Aldrich 366897) and d_8-FA-C20:4 (5 μg/mL, Cayman Chemicals 390010) was added. Upper aqueous phase (1/3 of total aqueous phase by mass) from Bligh and Dyer's extraction (Figure 1) containing polar carboxylic acids was used to resuspend the dried lipid extract, then mixed with derivatisation reaction base comprising 180 μL of 180 mM of EDC.HCl (Sigma-Aldrich E6383) and 7.5% (v/v) of pyridine (Sigma-Aldrich 360567) dissolved in methanol: isopropanol (3:7, v/v). For derivatisation of carboxylic acids, 180 μL of 300 mM 3-nitrophenylhydrazine hydrochloride (3-NPH.HCl, Sigma-Aldrich N21804) in methanol: water (7:3, v/v) was added to the samples, which were then vortexed and incubated at 600 rpm for 60 min at 37°C. At the end of the reaction, the samples were cooled at - 20° for 5 min, then centrifuged at 12,000 rpm for 2 min at 4°C. Clean supernatant was transferred to a new tube and dried in a SpeedVac under OH mode. The derivatised carboxylic acids were resuspended in methanol prior to MS analysis.

Organic acid analyses

Organic acids (including C2-C36 FFAs and polar carboxylic acids) were analyzed on a Jasper HPLC connected to Sciex 4500 MD system. Individual metabolites were separated on a Phenomenex Kinetex C18 column (100 × 2.1 mm, 2.6 μm) under gradient conditions with mobile phase A: 0.1% formic acid in acetonitrile: water (1:9, v/v) containing 2 mM ammonium acetate and mobile phase B: 0.1% formic acid in acetonitrile: methanol: isopropanol (2:1:2, v/v/v). The gradient started with 4% B that was maintained for 2 min, which underwent stepwise increases, first to 72% B over 13 min and further increased to 73% B over the next 3.5 min, then to 85% over 1.5 min and further to 86% over 1 min. From the 20th to 21.5th min, the gradient was increased from 86% B to 100% B, and was maintained at 100% B for 7.5 min, before returning to 4% B over 0.5 min and equilibrated for another 5.5 min before the next injection. Flow rate was at 300 μL/min and column temperature was at 40°C. Derivatised organic acids were analyzed under electrospray ionization (ESI) in the negative ion mode (TEM 500°C, GS1 = 45, GS2 = 35). SCFAs (C2-C6) and MCFAs (C7-C12) were quantitated using butyric-2,2-d_2-acid and octanoic acid-1-[13] C as internal standards, respectively. For LCFAs and VLCFAs, fully saturated FAs were quantitated using d_{31}-FA-C16:0 as internal standard, while d_8-FA-C20:4 served as internal standard for unsaturated FAs. Polar carboxylic acids comprising the TCA cycle were quantified using 2,2,3,3-d_4-succinic acid, d_4-fumaric acid, 2,2,4,4-d_4-citric acid, 2,3,3-d_3-malic acid, d_3-pyruvic acid and d_3-lactic as internal standards.

Lipidomics analyses

The portion of lipid extract for full lipidome analysis was resuspended in chloroform: methanol (1:1, v/v) and analyzed on a system comprising Jasper HPLC connected with Sciex Triple Quad 4500 MD. Individual lipid classes were separated with normal phase (NP)-HPLC using TUP-HB silica column (i.d. 150 × 2.1mm, 3 μm) under the following conditions, with mobile phase A comprising chloroform: methanol: ammonium hydroxide, 89.5:10:0.5 (v/v/v) and mobile phase B of chloroform: methanol: ammonium hydroxide: 100 mM ammonium acetate: water, 27:65:1:2.5 (v/v/v/v/v). Multiple reaction monitoring (MRM) transitions were set up for quantitative analyses of various lipid classes, as comprehensively reported in a preceding publication.[50] Lipids were analyzed in two injections under ESI positive ion mode and negative ion mode, respectively. In the positive ion mode, the gradient started with 35% B that was maintained for 3 min, which was increased to 50% B over 1 min and maintained for another 1 min, and further increased to 100% B over 0.5 min. The gradient was maintained at 100% B for 2.5 min, before returning to 35% B over 0.5 min and equilibrated for 1.5 min before the next injection. In the negative ion mode, the gradient started with 2% B that was maintained for 2 min, then increased to 35% B over 1 min, which was further increased to 55% B over 1 min and maintained for another 1 min, before increasing again to 100% B over 0.5 min. The gradient was maintained at 100% B for 2.5 min, before returning to 2% B over 0.5 min and equilibrated for another 1.5 min prior to the next injection. Flow rates were 300 μL/min and column temperature was at 30°C for analyses in both positive and negative ion modes. Ion source temperature was at 450°C, with GS1 = 45 and GS2 = 45. Individual lipid species were quantified by referencing to spiked internal standards, which included d_9-PC 32:0(16:0/16:0), PE 17:0/17:0, d31-PS, PA17:0/17:0, PG14:0/14:0, CL14:0/14:0/14:0/14:0, BMP 14:0/14:0, SL d18:1/12:0, LPC 17:0, LPI 17:1, LPA 17:0, LPS17:1, Cer d18:1/17:0, SM d18:1/12:0, S1P d17:1, Sph d17:1, GluCer d18:1/8:0, LacCer d18:1/8:0 and DAG 18:1/18:1-d5 from Avanti Polar Lipids, diC8-PI from Echelon Biosciences, Gb3 d18:1/17:0 and GM3 d18:1/18:0-d_3 from Matreya LLC, d_3-16:0 carnitine from Cambridge isotopes, as well as d_6-CE18:0 and TAG$(16{:}0)_3$-d_5 from CDN isotopes.

QUANTIFICATION AND STATISTICAL ANALYSIS

For determination of limit of detection (LOD) and limit of quantification (LOQ), calibration curves were generated using linear regression analysis by plotting signal intensity against nominal concentration. LOD was calculated as 3.3 times the standard error of the regression (residual standard error) divided by the slope of the calibration curve LOD = $3.3\sigma/S$, while LOQ = $10\sigma/S$. Changes in endogenous abundances of lipids between young and old mice was compared using Student's *t* test for individual tissue types, with statistical significance defined as $p < 0.05$. Differential lipid species that overlapped between different comparisons were visualized using an UpSet plot (generated with the R package UpSetR) and Venn diagrams (constructed with ggVennDiagram). Differential lipid patterns were illustrated as heatmaps using the ComplexHeatmap package. All analyses were conducted using R version 4.4.1 (R Foundation for Statistical Computing).

Cell Reports Methods, Volume *6*

Supplemental information

A multi-tiered workflow for examining organic acid profiles delineates tissue-specific changes in fatty acyl partitioning during aging

Zhiyang Zhou (周志阳), Chenyin Cao (曹晨音), Taochao Lu (陆陶超), Yuyuan Ruan (阮玉园), Bowen Li (李博文), Mingjun Cao (曹明君), Luyue Mo (莫璐月), Guanghou Shui (税光厚), and Sin Man Lam (林茜雯)

Supplemental Tables

Table S2. Percent recovery of representative organic acids from different subclasses in brain tissue matrix, related to Figure 3.

Mean % recoveries were calculated from averages of three independent experiments.

Class	*Representative species*	*% Recovery in brain tissue matrix*
SCFA	FA3:0	75.8
MCFA	FA14:0	85.1
LCFA	FA16:1(ω10)	89.1
OCFA	FA17:0	100.1
PUFA	FA20:4	116.4
	FA22:6	115.8
VLCFA	FA26:0	89.6
	FA28:0	93.4
	FA30:0	96.1
TCA	succinic acid	101.8
	fumaric acid	85.8
	malic acid	103.3
	alpha-ketoglutaric acid	111.8

SCFA: short-chain fatty acids; MCFA: medium-chain fatty acids; LCFA: long-chain fatty acids; OCFA: odd-chain fatty acids; PUFA: polyunsaturated fatty acids; VLCFA: very-long-chain fatty acids; TCA: tricarboxylic acids

Supplemental figures

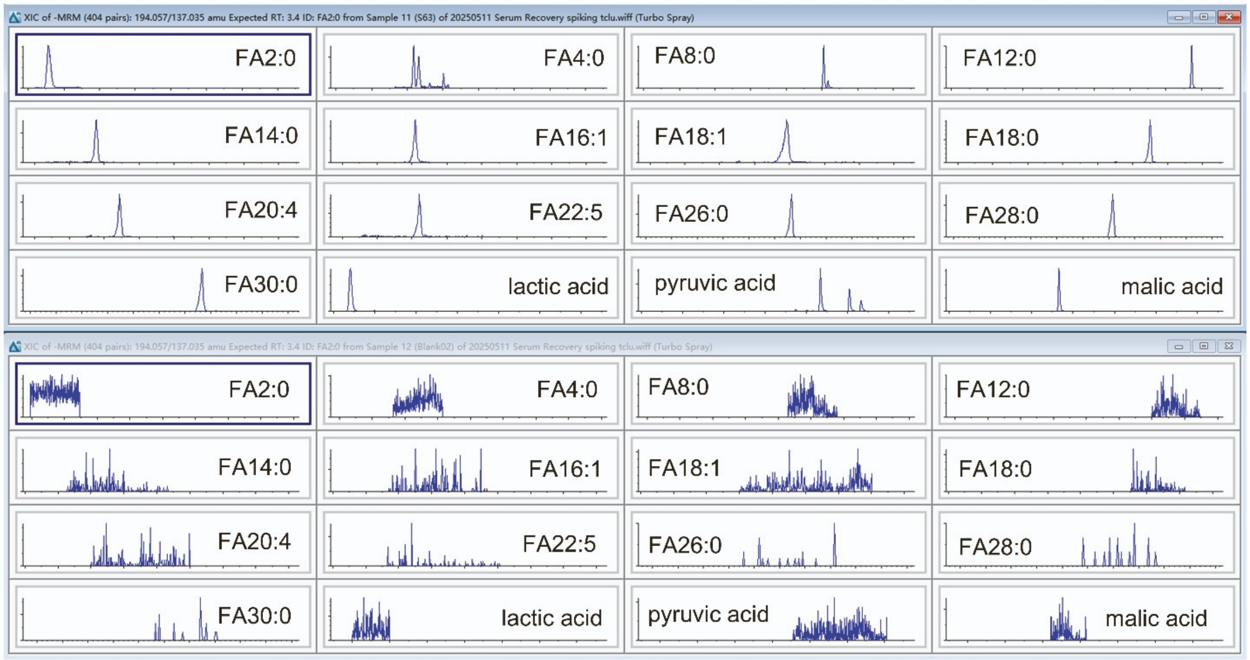

Figure S1. Carryover of individual organic acids under our liquid chromatographic gradient scheme, related to Figure 2. XICs of representative organic acids in reference standard mix (upper panel) and blank sample (lower panel) injected immediately after the reference standard mix showed negligible carryover under our liquid chromatographic gradient scheme**.**

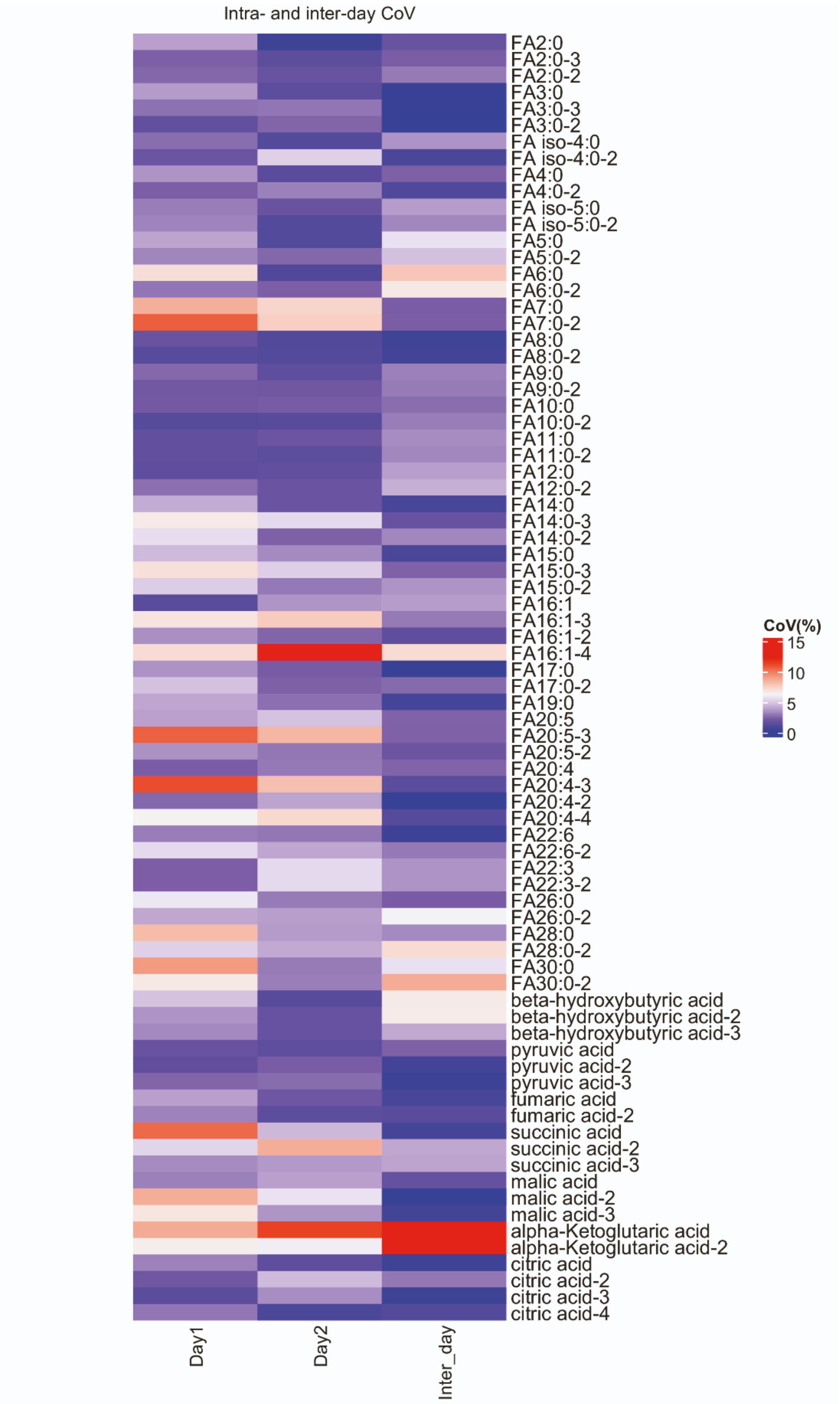

Figure S2. Intra-day and inter-day coefficient of variations, related to Figure 3. Heatmap summarizes the intra-day (three time-points on each day for two days, n=6 technical replicates) and inter-day (Day 1 vs Day 2, n=6 technical replicates) coefficient of variations (COVs) for individual organic acids quantified under our integrated methodology.
